# Designing functional hyaluronic acid-based hydrogels for cartilage tissue engineering

**DOI:** 10.1016/j.mtbio.2022.100495

**Published:** 2022-11-13

**Authors:** Min Wang, Zexing Deng, Yi Guo, Peng Xu

**Affiliations:** a Honghui Hospital, Xi'an Jiaotong University, Xi'an, 710000, China; b College of Materials Science and Engineering, Xi'an University of Science and Technology, Xi'an, 710054, China; c Shaanxi Key Laboratory of Brain Disorders, Shaanxi Key Laboratory of Ischemic Cardiovascular Disease, Institute of Basic and Translational Medicine, Xi'an Medical University, Xi'an, 710021, China

**Keywords:** Hyaluronic acid, Hydrogel, Functional modification, Cartilage tissue engineering

## Abstract

Damage to cartilage tissues is often difficult to repair owing to chronic inflammation and a lack of bioactive factors. Therefore, developing bioactive materials, such as hydrogels acting as extracellular matrix mimics, that can inhibit the inflammatory microenvironment and promote cartilage repair is crucial. Hyaluronic acid, which exists in cartilage and synovial fluid, has been extensively investigated for cartilage tissue engineering because of its promotion of cell adhesion and proliferation, regulation of inflammation, and enhancement of cartilage regeneration. However, hyaluronic acid-based hydrogels have poor degradation rates and unfavorable mechanical properties, limiting their application in cartilage tissue engineering. Recently, various multifunctional hyaluronic acid-based hydrogels, including alkenyl, aldehyde, thiolated, phenolized, hydrazide, and host–guest group-modified hydrogels, have been extensively studied for use in cartilage tissue engineering. In this review, we summarize the recent progress in the multifunctional design of hyaluronic acid-based hydrogels and their application in cartilage tissue engineering. Moreover, we outline the future research prospects and directions in cartilage tissue regeneration. This would provide theoretical guidance for developing hyaluronic acid-based hydrogels with specific properties to satisfy the requirements of cartilage tissue repair.

## Graphical abstract

**Figure:**
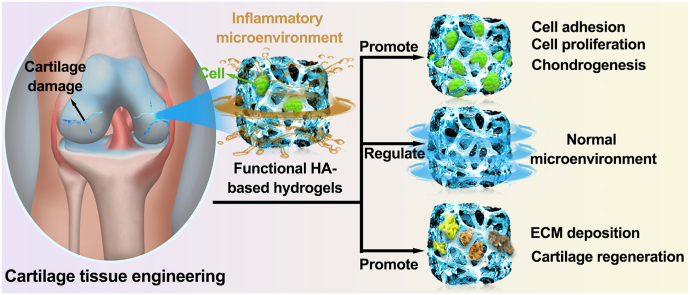

## 1 Introduction

Articular cartilage is a special soft tissue composed of chondrocytes and extracellular matrix (ECM) and is located on the surface of bone joints. It plays an important role in maintaining lubrication and joint movement [[1], [2], [3]]. Articular cartilage damage due to trauma, congenital anomalies, and diseases can often cause joint pain and motor dysfunction in patients [[4], [5], [6]]. Owing to the unique tissue structure of the articular cartilage, its repair and treatment after an injury has significant limitations and challenges; the limiting factors include the lack of blood vessels, nerves, and lymph, high ratio of ECM to cells, and elevated expression of proinflammatory factors [[7], [8], [9], [10], [11]]. A continuous provision of bioactive factors as well as the inhibition of the inflammatory response at the articular cartilage injury site is thus required.

Current treatment strategies for articular cartilage injury primarily rely on surgical treatments such as microfractures, subchondral drilling, and autologous chondrocyte transplantation. However, these strategies often have certain limitations, including infliction of trauma, low efficacy, and the formation of postoperative fibrocartilage, which can damage joint function [[12], [13], [14]]. Compared with the aforementioned strategies, biomedical hydrogels can provide a biomimetic ECM similar to natural cartilage. Hydrogels are a class of three-dimensional hydrophilic polymer networks with water in their matrix that can be loaded with cells, drugs, and bioactive molecules and maintain their activity; they have been widely used in cartilage damage repair [[15], [16], [17], [18]]. In cartilage tissue engineering, ideal hydrogels should have a biomimetic cartilage ECM to build an ecological microenvironment and multifunctional properties, including maintenance of cell viability and phenotype, reduction of fibrocartilage generation, suitable degradation rate, excellent biocompatibility, sustained release of drugs and growth factors, and reconstruction of the bone–cartilage interface [19].

**Fig. 1:**
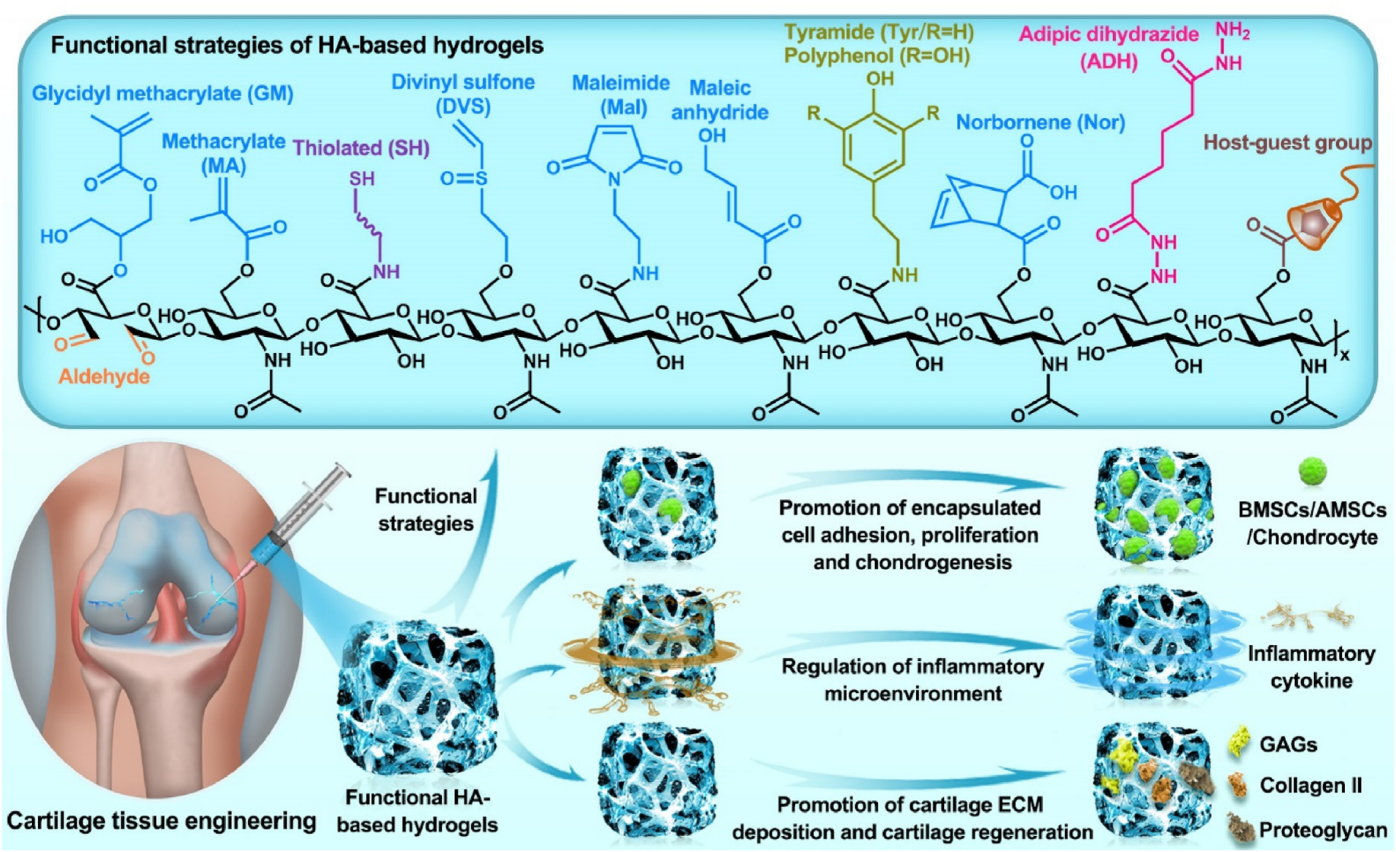
Synthesis and biological function of HA-based hydrogel for cartilage tissue engineering.

**Fig. 2:**
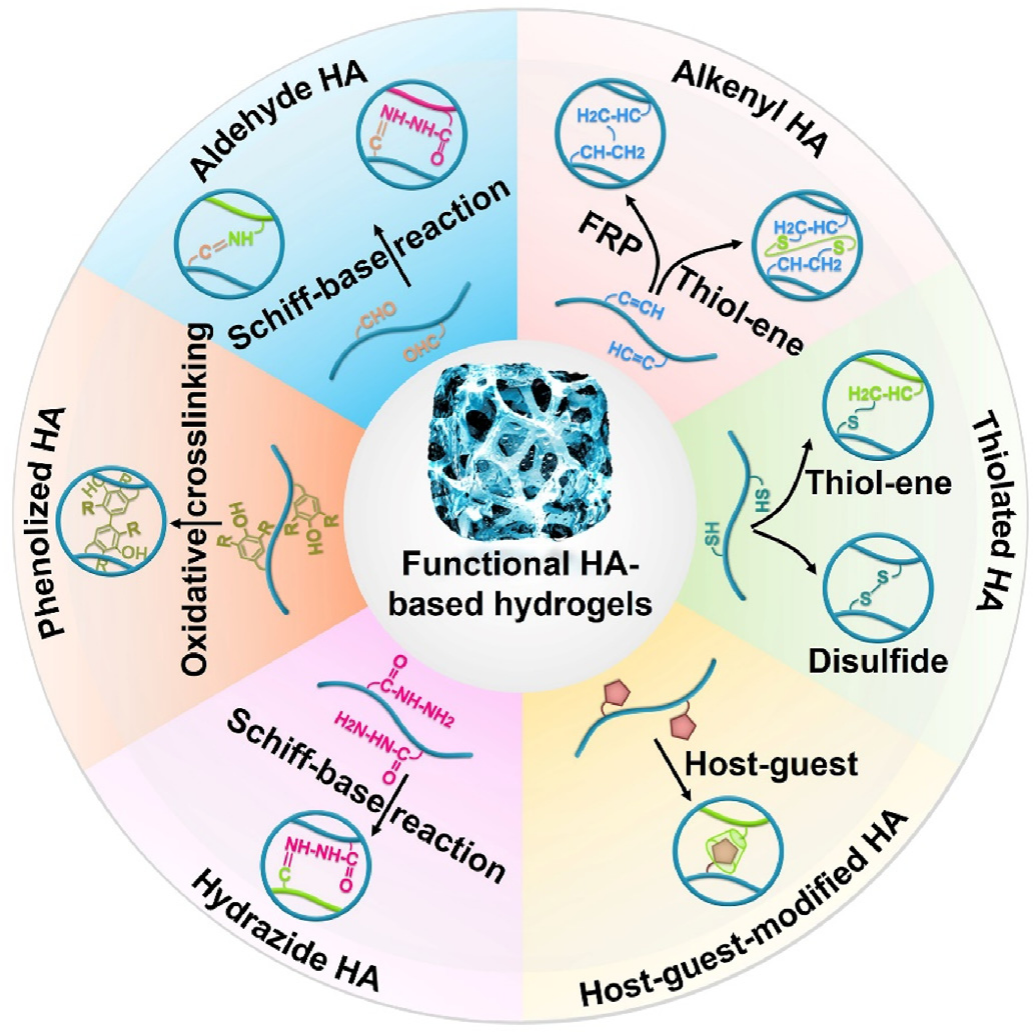
Functional modification of HA-based hydrogels.

Polysaccharides have significant application potential in cartilage tissue engineering because of their similar structures to that of cartilage ECM components, high rheology and water retention, excellent biocompatibility, and antioxidant and anti-inflammatory properties [20]. Hyaluronic acid (HA) is a polysaccharide composed of alternating d-glucuronic acid and *N*-acetylglucosamine, which are naturally present in cartilage and synovial fluid [21,22]. Compared with other polysaccharides, HA affects the regulation of cartilage function and repair of cartilage damage in many ways. Previous studies have demonstrated that HA can improve the lubricity of cartilage boundaries, regulate inflammation at cartilage lesions, promote cell adhesion and proliferation, and ameliorate cartilage ECM deposition and cartilage regeneration, all of which have excellent application prospects in cartilage tissue engineering [[23], [24], [25]]. Notably, HA can recognize the HA receptor (cluster of differentiation 44, CD44) to protect cartilage and participate in the regeneration of cartilage ECM during cartilage repair [20]. Therefore, HA-related commercial products, such as Artz®, Cingal®, Durolane®, Hyalgan®, Monovisc®, Orthovisc®, Sinovial®, Supartz®, and Synvisc®, have been widely used for cartilage-related diseases. Currently, most related clinical studies are still in progress (Table 1) (www.clinicaltrials.gov). In addition, functional modification of HA-based hydrogels can effectively promote cartilage repair and regeneration by enhancing the adhesion, proliferation, and chondrogenesis of encapsulated stem cells and chondrocytes, regulating the inflammatory microenvironment at the cartilage injury site, and promoting cartilage ECM deposition, such as glycosaminoglycan (GAG), collagen II, and proteoglycans [20,26] (Fig. 1). In recent years, functional modification based on HA hydrogels in cartilage tissue engineering has primarily focused on the modification of three reactive functional groups in the molecular structure of HA: 1) esterification and acylation of carboxyl groups, 2) esterification and divinyl sulfone (DVS) crosslinking of primary hydroxyl groups, and 3) oxidation reaction of secondary hydroxyl groups [27,28] (Fig. 2).

**Table 1:** Clinical trials of HA in cartilage related disease (Source: www.clinicaltrials.gov).

Gov identifier	Sponsor	Phase	Disease	Interventions	Purpose
NCT03101163	KLSMC Stem Cells, Inc.	Phase 2	Articular Cartilage Disorder	Peripheral blood stem cells combined with HA (PBSCs/HA)	Evaluating the therapeutic effects between PBSCs/HA and standard treatment after subchondral drilling surgery
NCT00988091	Ferring Pharmaceuticals	Phase 3	Knee osteoarthritis	1.2% sodium hyaluronate	Evaluating the therapeutic effects of 1.2% sodium hyaluronate through intra-articular injections
NCT01319461	Med Pharma Co., Ltd.	Phase 3	Knee osteoarthritis	Hyalgan® (sodium hyaluronate); Sterile normal saline	Evaluating the therapeutic effects between Hyalgan® and sterile normal saline through intra-articular injections
NCT01295580	Bioventus LLC	Not Applicable	Knee osteoarthritis	Artz® (HA); Durolane® (HA stabilized)	Evaluating the safety and therapeutic effects between Artz® and Durolane® through intra-articular injections
NCT00556608	IBSA Institut Biochimique SA	Phase 4	Knee osteoarthritis	Sinovial® (sodium hyaluronate); Synvisc® (Hylan G-F 20)	Evaluating the safety and therapeutic effects between Sinovial® and Synvisc® through intra-articular injections
NCT02211521	Samsung Medical Center	Phase 3	Knee osteoarthritis	HA; Autologous platelet-rich plasma	Evaluating the therapeutic effects between HA and autologous platelet-rich plasma through intra-articular injections
NCT04165902	Taipei Medical University	Phase 4	Knee osteoarthritis	Steroid plus HA; Dextrose plus HA	Evaluating the additional therapeutic effects of steroid and dextrose to HA through injection
NCT03062787	Pharmascience Inc.	Not Applicable	Knee osteoarthritis	Cingal® (HA plus triamcinolone hexacetonide); Monovisc® (sodium hyaluronate)	Evaluating the early effects between Cingal® and Monovisc® through intra-articular injections
NCT03211650	University of Milan	Phase 4	Knee osteoarthritis	HA and platelet-rich plasma (PRP) combination	Evaluating the therapeutic effects of HA and PRP combination through intra-articular injections
NCT01557868	American Orthopaedic Society for Sports Medicine	Phase 4	Knee osteoarthritis	Synvisc® (Hylan G-F 20); 1% sodium hyaluronate	Developing the algorithm to predict the therapeutic effects of HA products through intra-articular injections
NCT02984228	Hospital for Special Surgery, New York	Phase 4	Glenohumeral osteoarthritis	HA; Platelet-rich plasma (PRP)	Evaluating the therapeutic effects between HA and PRP through ultrasound-guided injections
NCT00918736	Kaohsiung Veterans General Hospital	Phase 2	Ankle osteoarthritis	Sodium hyaluronate	Evaluating the therapeutic effects of sodium hyaluronate through three weekly intra-articular injections
NCT04204278	Anika Therapeutics, Inc.	Not Applicable	Ankle osteoarthritis	Monovisc® (sodium hyaluronate)	Evaluating the therapeutic effects of Monovisc® through single injections
NCT00436969	DePuy Mitek	Phase 3	Shoulder osteoarthritis	Orthovisc® (HA); Corticosteroid	Evaluating the therapeutic effects between Orthovisc® and corticosteroid through injections
NCT00479687	Bioventus LLC	Not Applicable	Shoulder osteoarthritis	Supartz® (HA); Phosphate buffered saline (PBS)	Evaluating the therapeutic effects between Supartz® and PBS through injections

In this paper, we discuss the functional molecular structure modification of HA-based hydrogels primarily containing alkenyl, aldehyde, thiolated, phenolized, hydrazide, and host–guest groups. We summarize their application in cartilage tissue engineering, and introduce the research status of HA-based hydrogels as 3D printing bioinks in cartilage engineering. We further elucidate the capacities and merits of different functionalized HA-based hydrogels to guide their research in cartilage tissue engineering. Finally, we highlight the advantages and challenges of current HA-based hydrogels for cartilage tissue-engineering applications.

## 2 Alkenyl HA-based hydrogels for cartilage tissue engineering

**Fig. 3:**
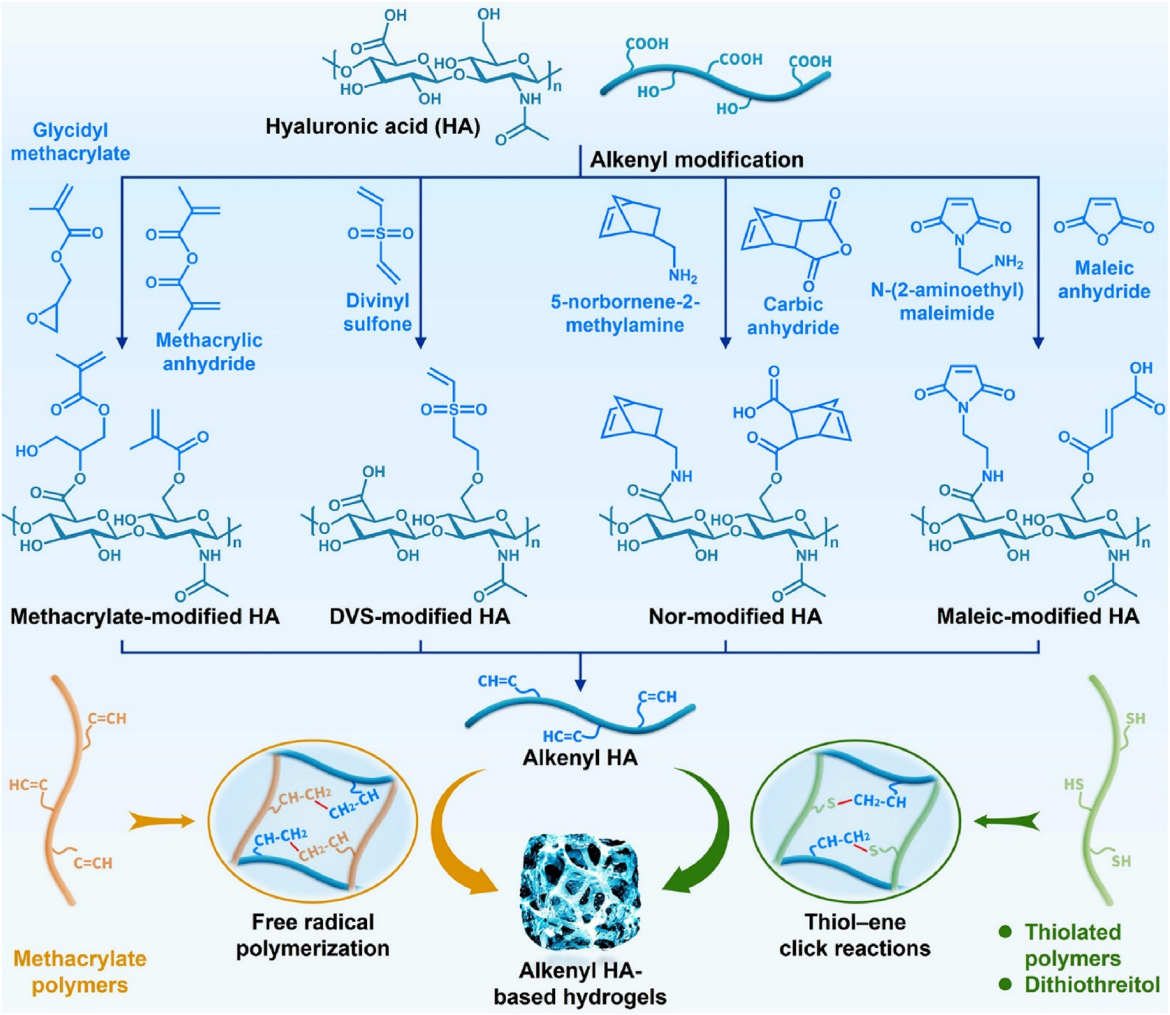
Synthesis of alkenyl HA-based hydrogels for cartilage tissue engineering.

Alkenyl HA-based hydrogels have been widely studied in cartilage tissue engineering because they can be prepared easily by double bonds taking part in simple chemical reactions, such as self-polymerization through free-radical polymerization (FRP) reactions and click chemistry with sulfhydryl groups. The reactions often are fast speeds, occur in mild conditions, and exhibit desirable adaptability and high directional selectivity (Fig. 3 and Table 2).

**Table 2:** Alkenyl HA-based hydrogels for cartilage tissue engineering.

Hydrogels	Components	Physicochemical properties	Biofunctions	Ref.
Methacrylated HA (HA-MA)	HA; methacrylic anhydride (MA)	Viscoelasticity; controlled pore size and degradation rate	Encapsulating cells; promoting MSCs proliferation, migration and chondrogenesis; promoting ECM deposition	[[30], [31], [32], [33], [34], [35]]
HA-MA/MeLAHA	HA, MA, MeLAHA	Hydrolytic degradation and enzymatic degradation	Promoting MSCs chondrogenesis and ECM deposition; ensuring stability of hydrogel scaffold	[36,37]
HA-MA/MMP7	HA, MA, matrix metalloproteinase 7	Tunable mechanical properties, swelling performance and degradation rates	Promoting MSCs chondrogenesis	[38,39]
HA-MA/Gel-MA	HA, MA, gelatin,	Improving the mechanical properties	Enhancing cell proliferation, aggregation and chondrogenesis; maintaining the chondrocyte phenotype; enhancing deposition and distribution of ECM; accelerating cartilage repair	[[40], [41], [42], [43], [44]]
HA-MA/Gel-MA/BC	HA, MA, gelatin, bacterial cellulose	Improving the mechanical properties and printing fidelity	Facilitating chondrocyte proliferation and protein expression	[45]
HA-MA/Gel-MA/AFnSi	HA, MA, gelatin, acrylate-functionalized nanosilica	Controllable pore sizes, swelling ratios and mechanical properties	Promoting the chondrogenic gene expression and ECM deposition	[46]
HA-MA/CNF	HA, MA, methacrylated cellulose nanofibers	Enhanced mechanical properties; decent restorability	Enhancing BMSCs proliferation and chondrogenesis; accelerating full-thickness cartilage repair	[48]
HA-MA/CF	HA, MA, chondrogenic fibrinogen	Ameliorating the compressive modulus	Enhancing BMSCs proliferation and early chondrogenesis;	[49]
HA-MA/ELP@ZnO	HA, MA, elastin-like polypeptide, zinc oxide	Adjustable mechanical strength, elasticity, and adhesion	Enhancing cell proliferation and migration; negligible inflammatory response; antibacterial ability	[50]
HA-MA/ROS-HP/PLGA	HA, MA, ROS-HP, poly (lactide-co-glycolide)	Suitable compressive modulus; good ROS scavenging ability	Regulating inflammation; promoting ECM deposition; accelerating hyaline cartilage regeneration and integration with surrounding tissues	[51]
HA-MA/F127DA	HA, MA, pluronic F127 diacrylate	Low swelling ratio; excellent stiffness, toughness and self-recovery	Enhancing thyroid cartilage regeneration of rabbit larynx	[52]
HA-MA/PRP	HA, MA, platelet-rich plasma	Suitable mechanical properties	Promoting ECM deposition and hyaline cartilage regeneration	[54]
HA-MA/SA/KGN-loaded PLGA	HA, MA, sodium alginate, kartogenin, poly (lactide-co-glycolide)	Microporous; promoting cells and tissues infiltration; sustained release of KNG	Regulating the inflammatory microenvironment; promoting migration and chondrogenesis of BMSCs; accelerating cartilage repair	[55,56]
HA-GM	HA, glycidyl methacrylate	Highly interconnected macroporous network; compression-recovery property	Maintaining the activity and phenotype of chondrocytes; promoting chondrocytes proliferation and ECM deposition	[57]
HA-DVS	HA, divinyl sulfone	Viscoelasticity; enzymatic degradation	Inducing the chondrogenic differentiation of AMSCs	[61]
HA-DVS/Li-BGF	HA, DVS, lithium bioactive glass fibers	Improving the elastic modulus	Promoting chondrocytes proliferation and chondrogenic behavior	[62]
HA-DVS/Gel-HS	HA, DVS, thiolated gelatin	Providing bioinspired microenvironment	Inhibiting vascularization and hypertrophy; promoting cartilage repair	[63]
HA-Nor/DTT	HA, 5-norbornene-2-methylamine, dithiothreitol	Controllable compressive moduli and CD44 binding	Promoting the deposition and distribution of ECM; promoting the chondrogenic differentiation of BMSCs	[[68], [69], [70], [71]]
HA-Nor/DTT	HA, carbic anhydride, dithiothreitol	Simple synthesis; controllable gelation properties, swelling and transmittance	Good cytocompatibility; encapsulating cells	[67]
HA-Mal/GelMA	HA, maleimide, gelatin, MA	Adjustable mechanical properties	Supporting the proliferation and chondrogenesis of BMSCs	[74]
HA-Mal/DTT	HA, maleimide, dithiothreitol	Adjustable mechanical strength	Maintaining BMSC stemness (lower strength), promoting BMSCs chondrogenesis (higher strength)	[75]
HA-Mal/PEG-SH	HA, maleic anhydride, thiol-terminated polyethylene glycol	High substitution degree; rapid gelation; regulatable degradation rate	Good cytocompatibility	[76]

### 2.1 Methacrylate HA-based hydrogels

Methacrylic anhydride (MA) has a highly reactive anhydride group and photocrosslinkable C=C double bonds, which can be easily grafted to natural polymers through the reaction of anhydride groups with amino groups or hydroxyl groups; moreover, functional polymers can be utilized to prepare hydrogels through photopolymerization [29]. Notably, an *in situ* photocrosslinkable methacrylate HA-based hydrogel with good viscoelasticity was prepared using esterification between the hydroxyl group of HA and the anhydride group of MA, which not only facilitated the encapsulation of cells but also reduced the mechanical stimulation of surrounding tissues [30]. The pore size, HA concentration, crosslinking density, and degradation rate of HA-MA-based hydrogels impact their application in cartilage tissue engineering [[31], [32], [33], [34], [35]]. Particularly, compared with other pore sizes, HA-MA-based hydrogels with medium pore sizes (200–250 ​μm) demonstrated the best promotion of endothelial cell proliferation and migration and exhibited the best vascularization behavior for cartilage repair [31]. Moreover, an increased concentration of HA in HA-MA-based hydrogels can enhance chondrogenesis and ECM deposition of encapsulated mesenchymal stem cells (MSCs), but it might cause the uneven distribution of the ECM [32,33]. In addition, the low crosslinking density (1% at 15 ​min exposure or 5% at 5 ​min exposure) of HA-MA (∼29% methacrylate modification)-based hydrogels can promote cartilage differentiation, whereas an increase in crosslinking density (5% at 15 ​min exposure) reduces the deposition and uniform distribution of cartilage ECM and induces hypertrophy and mineralization of MSCs [34]. In addition, the degradation rate of hydrogels plays an important role in cartilage repair. When the degradation rate is high, it is not beneficial for the retention of ECM; whereas when the degradation rate is low, it inhibits the formation of cartilage tissue [[35], [36], [37]]. Compared with collagen-based hydrogels, HA-MA-based hydrogels can maintain the proliferation of bone marrow MSCs (BMSCs) and the production of cartilage-related ECM in the later stages of cartilage tissue engineering because of their relatively stable physical microenvironment. However, the cartilage-inducing activity of HA-MA-based hydrogels cannot compete with that of collagen-based hydrogels in the early stages of cartilage tissue engineering [35]. To optimize the degradation rate of HA-MA-based hydrogels *in vivo*, *Burdick* et al. prepared a hybrid hydrogel using HA-MA and a novel HA macromer (MeLAHA, HA macromers with methacrylate modification and *ε*-caprolactone) with hydrolytic and enzymatic degradation performance, which promoted the chondrogenesis of MSCs by controlling the degradation property of the hydrogel. This is because faster degradation (complete degradation within 7 days) of MeCLHA provided void spaces for the deposition of ECM, and slower degradation (∼40% degradation in 56 days) of HA-MA ensured the stability of the hydrogel scaffold [36,37]. In addition, *Tsanaktsidou* et al. prepared another HA-MA-based hydrogel with two different crosslinking mechanisms, including FRP reaction and matrix metalloproteinase 7 (MMP7)-peptide-induced thiol-ene click reactions, which have tunable mechanical properties, swelling performance, and degradation rates. Interestingly, HA-MA-based hydrogels prepared using the MMP7-peptide in Dulbecco's modified Eagle's medium (DMEM) are beneficial for the chondrogenesis of human MSCs (hMSCs) because of their appropriate storage modulus of approximately 12 ​kPa [38]. *Tsanaktsidou* et al. further prepared a chondroitin sulfate (CS)-biofunctionalized HA-MA hydrogel by crosslinking the MMP7-peptide, which effectively enhanced hyaline cartilage regeneration by upgrading cartilage-related gene expression, GAG deposition, and chondrocyte cluster arrangement [39]. Therefore, the aforementioned reports provide significant guidance for the design and regulation of HA-MA-based hydrogels in cartilage tissue engineering.

In addition to the pore size, HA concentration, crosslinking density, and degradation rate of HA-MA-based hydrogels, mechanical properties and swelling rate influence cartilage tissue engineering. Studies have reported that suitable mechanical properties and a low swelling rate of HA-MA-based hybrid hydrogels can be achieved by incorporating natural polymers, which can effectively promote cartilage repair. For instance, *Levett* et al. prepared a hybrid hydrogel (HA-MA/Gel-MA) using HA-MA and gelatin (porcine skin) methacrylamide (Gel-MA) *via* photoinduced polymerization. The HA-MA component significantly improved the mechanical properties of the hydrogel, maintained the chondrocyte phenotype with rounded cell morphologies, and enhanced the deposition and distribution of ECM components, including collagen II and aggrecan, to accelerate cartilage formation. In addition, they incorporated CS methacrylate into an HA-MA/Gel-MA hybrid hydrogel to improve its mechanical properties, which enhanced chondrogenesis as well as the spatial distribution of the ECM [40,41]. Moreover, *Lin* et al. further demonstrated that the HA-MA/Gel-MA hybrid hydrogel can accelerate cartilage repair *in vivo* by promoting the chondrogenesis of hMSCs and the production of GAG when the concentration ratio of HA-MA and Gel-MA is 1:9 [42]. Interestingly, *Wang* et al. used HA-MA/Gel-MA hybrid hydrogels to encapsulate chondrocyte spheroids prepared using the hanging drop method for cartilage repair. Compared with chondrocytes, chondrocyte spheroids can form structures similar to native cartilage tissue, which can enhance cell proliferation and aggregation, maintain the cellular phenotype, and promote the deposition of abundant ECM [43]. Furthermore, compared with porcine-derived Gel-MA, the HA-MA/Gel-MA hybrid hydrogel constructed using bovine-derived Gel-MA and HA-MA through irgacure 2959-induced photocrosslinking (365 ​nm) exhibited the best similarity to native articular cartilage after 28 days of chondrocyte cultivation [44]. The mechanical properties of HA-MA/Gel-MA hybrid hydrogels can be further optimized by incorporating organic polymers or inorganic nanoparticles [45,46]. For example, *Sang* et al. incorporated bacterial cellulose (BC) into an HA-MA/Gel-MA hybrid hydrogel, which not only improved the mechanical properties and printing fidelity of the hydrogel but also facilitated chondrocyte proliferation and specific protein expression [45]. *Nedunchezian* et al. used acrylate-functionalized nanosilica (AFnSi) to crosslink HA-MA/Gel-MA hybrid hydrogels, and the obtained hydrogels exhibited controllable pore sizes, swelling ratios, and mechanical properties. Moreover, the hydrogels could promote the gene expression of chondrogenic markers, including SOX9, proteoglycan, and collagen II, in human adipose-derived MSCs (AMSCs) and increase the formation of sulfated GAG (sGAG) and collagen II [46].

In addition to Gel-MA, the incorporation of other natural polymers into HA-MA-based hydrogel matrices can optimize their mechanical properties and promote the chondrogenesis of cells [47]. For example, *Zhao* et al. fabricated a nanocomposite hydrogel (HA-MA/CNF) through the co-crosslinking of HA-MA and methacrylated cellulose nanofibers (CNFs); the HA-MA/CNF hydrogel exhibited enhanced mechanical properties with a compressive modulus of ∼0.46 ​MPa, a compressive strength of ∼0.198 ​MPa, and satisfactory restorability. Moreover, biological experiments have indicated that the nanocomposite hydrogel can have positive effects on the proliferation and chondrogenesis of BMSCs as well as accelerate full-thickness cartilage repair *in vivo* [48]. *Snyder* et al. developed a hybrid hydrogel comprising HA-MA and chondrogenic fibrinogen to ameliorate the compressive modulus of HA-MA-based hydrogels, which provided a suitable microenvironment for the proliferation and delivery of BMSCs as well as increased expression of SOX9 and decreased collagen I, suggesting that it was favorable for early chondrogenesis [49]. *Sani* et al. prepared a bioactive hydrogel (HA-ELP) using HA-MA and elastin-like polypeptide (ELP), which had adjustable mechanical properties including mechanical strength, elasticity, and adhesion. Meanwhile, the HA-ELP hydrogel supported cell proliferation and migration but had a negligible inflammatory response *in vivo*. Moreover, the HA-ELP hydrogel exhibited high antibacterial activity against drug-resistant bacteria after doping with zinc oxide (ZnO) nanoparticles, demonstrating good application potential in tissue engineering such as cartilage repair [50]. In addition, compared with natural polymers, synthetic polymers have also been reported to optimize mechanical properties and endow multifunctionality to HA-MA-based hydrogels [51,52]. *Wu* et al. designed a novel hybrid scaffold by filling an antioxidant hydrogel composed of HA-MA and reactive oxygen species (ROS)-responsive hyperbranched polymers (ROS-HP) into a poly (lactide-co-glycolide) (PLGA) scaffold with radial orientation. The hybrid scaffold had a suitable compressive modulus (1.5 ​MPa), good ROS-scavenging ability, and ability to regulate inflammation to accelerate hyaline cartilage regeneration. Moreover, the incorporation of hydrogel had a positive impact on the deposition of GAG and collagen II to integrate with surrounding tissues for neocartilage [51]. *Ren* et al. developed a stiff micelle-crosslinked hydrogel using HA-MA and Pluronic F127 diacrylate (F127DA), which exhibited a low swelling ratio and excellent mechanical properties, including stiffness, toughness, and self-recovery. The built hydrogel was further used in thyroid cartilage defects of rabbit larynx, and animal studies showed that the hydrogel effectively contributed to cartilage regeneration [52].

**Fig. 4:**
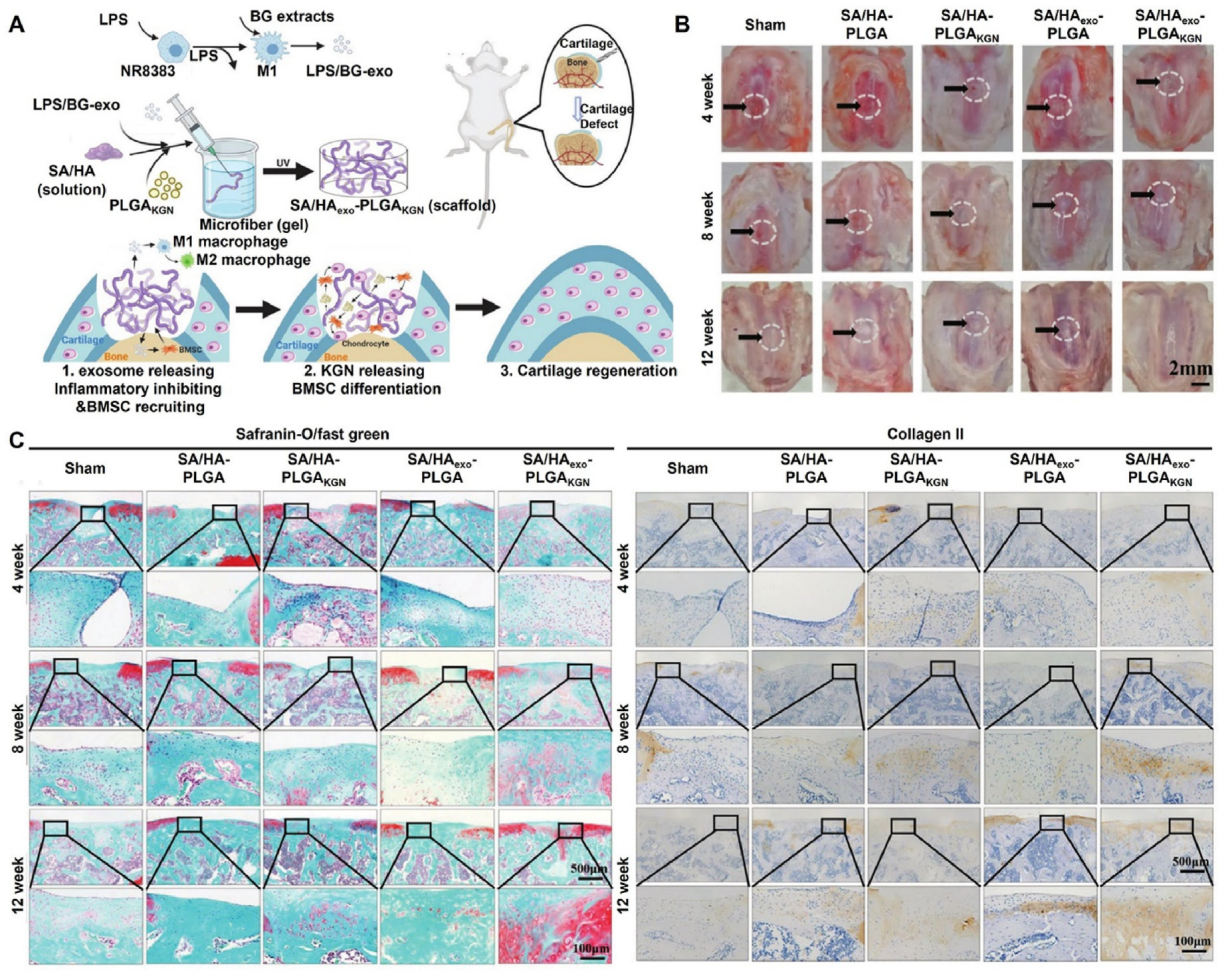
Schematic illustration of the fabrication of SA/HAexo-PLGAKGN scaffold for cartilage regeneration. (A) Preparation of SA/HA_exo_-PLGA_KGN_ scaffold and its biological function; (B) gross images of the cartilage defects in different groups at weeks 4, 8, and 12 after surgery; (C) immunohistochemical staining images of cartilage defects in different groups after surgery for 4, 8, and 12 weeks [56]; Copyright 2022, John Wiley and Sons.

Cartilage can generally be divided into three types: hyaline, elastic, and fibrocartilage. Notably, the regeneration of hyaline cartilage is crucial in articular cartilage repair [53]. *Yan* et al. encapsulated platelet-rich plasma (PRP) in an HA-MA hydrogel to increase the deposition of GAG and collagen II. In addition, the regeneration of hyaline cartilage was verified without hypertrophic cartilage formation in a porcine femoral condyle cartilage injury model [54]. *Shi* et al. incorporated small molecule kartogenin (KGN)-loaded nanoparticles into HA-MA hydrogels to recruit and facilitate chondrogenesis of different endogenous cells (BMSCs, synovium-derived MSCs (SMSCs), and chondrocytes). Furthermore, the hydrogel could fill the defect and release KGN continuously to promote the formation of hyaline cartilage in a cartilage defect model of rabbit knee joints [55]. To avoid poor cell and tissue infiltration caused by nanoporous hydrogels, *Ma* et al. prepared a microporous hydrogel using HA-MA, sodium alginate (SA), and KGN-loaded PLGA microspheres using photopolymerization after crosslinking HA-MA/SA into microfibers to effectively promote the infiltration of cells and tissues. In addition, the hydrogel can gradually release exosomes and KGN-loaded PLGA microspheres, which can regulate the inflammatory microenvironment, enhance the migration of endogenous BMSCs, induce chondrogenesis of BMSCs, and promote cartilage repair (Fig. 4) [56].

In addition to the HA-MA hydrogel, *He* et al. fabricated a shape-memory-capable cryogel (HA-GM) using HA and glycidyl methacrylate (GM). Compared with HA-GM hydrogels, HA-GM cryogels with a highly interconnected macroporous network structure and compression-recovery property can provide a suitable microenvironment to maintain the activity and phenotype of chondrocytes during injection, as well as promote the proliferation of chondrocytes and the deposition of cartilage ECM [57]. *Hsieh* et al. developed a biomimetic scaffold using HA-GM, methoxy poly (ethylene glycol)-block-poly (*ε*-caprolactone) (mPEG-PCL), and hydroxyapatite loaded with transforming growth factor-*β*1 (TGF-*β*1) for the regeneration of hyaline cartilage and the ingrowth of bone tissue into the scaffold after implantation [58].

### 2.2 Divinyl sulfone-modified HA-based hydrogels

Recently, DVS has attracted considerable interest because of its hydrolysis resistance and broad-spectrum reactivity. It can modify biochemical molecules by reacting with sulfhydryl, amino, and hydroxyl groups under certain conditions [59,60]. *Mondal* et al. prepared an injectable hydrogel (HA-DVS) using HA and different concentrations of DVS *via* the reaction between hydroxyl and vinyl sulfone groups. The HA-DVS hydrogel exhibited viscoelasticity and enzymatic degradation ability and could induce the chondrogenesis of AMSCs [61]. *Riveiro* et al. further significantly improved the elastic modulus of HA-DVS hydrogels by doping them with lithium bioactive glass fibers (Li-BGF), which promoted the proliferation and chondrogenic behavior of chondrocytes [62]. *Feng* et al. developed a BMSC-laden bio-inspired hydrogel using HA-DVS and thiolated gelatin (Gel-HS) to enhance cartilage repair. The obtained hydrogels provided a good microenvironment for cell loading and minimally invasive delivery, protecting and maintaining the viability of BMSCs, and demonstrating positive effects on proliferation, chondrogenesis, and self-assembly into cartilage-like tissues, ultimately through interconnected cells. In addition, hydrogels can inhibit vascularization and hypertrophy, and contribute to cartilage repair [63]. HA-DVS-based hydrogels exhibited promising potential for the treatment of knee osteoarthritis (OA) in clinical trials. *Park* et al. evaluated the therapeutic effect of a novel HA-DVS hydrogel (YYD302) produced by biological fermentation on human knee OA after intra-articular injection. Compared with Synovian®, an HA-based hydrogel crosslinked with 1,4-butanediol diglycidyl ether, YYD302, could reduce the weight-bearing pain of patients after a single injection for 12 weeks and exhibited a better treatment effect at 2 weeks. In addition, YYD302 demonstrated good biosafety in local and systemic adverse drug reaction (ADR) evaluations [64]. *In* et al., the efficacy and safety of a single intra-articular injection of YYD302 in patients with knee OA were evaluated. Compared with phosphate-buffered saline, YYD302 effectively reduced pain and improved knee function, and the efficacy of a 2 ​mL dose was better than that of a 3 ​mL dose [65].

### 2.3 Norbornene-modified HA-based hydrogels

Norbornene (Nor)-thiol photocurable hydrogels, another type of material system with high application value in tissue engineering, are typically prepared using Nor-modified macromolecules (HA, gelatin, and polyethylene glycol (PEG)) and sulfhydryl-containing linkers. Such hydrogels have been demonstrated to have tunable biophysical and biochemical properties and good biocompatibility [66]. Compared with the widely studied HA-MA-based hydrogels, nor-modified HA (HA-Nor)-based hydrogels have attracted significant interest in biomedical research because of their low photoinitiator requirements and more stable and tailored structures [67]. *Galarraga* et al. prepared HA-Nor using HA and 5-norbornene-2-methylamine using a two-step method, including ion-exchange resins and catalytic reaction, and further crosslinked HA-Nor with dithiothreitol (DTT) to prepare hydrogels (HA-Nor-DTT) with different compressive moduli. Compared with stiffer (∼6–60 ​kPa) hydrogels, softer hydrogels (∼2 ​kPa) can provide a favorable local microenvironment to promote the deposition and distribution of ECMs, which promotes the chondrogenesis of encapsulated BMSCs. Moreover, the doping of polycaprolactone (PCL) microfibers produced by melt-electrowriting into softer hydrogels can solve the problems of poor initial mechanical properties and poor stability and promote neocartilage regeneration and integration [68]. The HA-Nor-DTT hydrogel has been used as a 3D bioprinting ink for cartilage tissue engineering [69]. *Vega* et al. further developed a combinatorial hydrogel platform with a biochemical gradient using an HA-Nor-DTT hydrogel and monothiolated peptides, which can be used to analyze early gene expression and long-term cartilage ECM of encapsulated MSCs. This platform can function as a scalable high-throughput technique for screening 3D cellular microenvironments [70]. Subsequently, to elucidate the influence of HA modification on CD44 binding, *Kwon* et al. evaluated the interaction of HA-Nor or HA-MA with different degrees of modification of CD44 and the effect on chondrogenesis of encapsulated MSCs. The results showed that the degree, type, and site of HA modification affected the binding of CD44. Low (∼10%) and moderately (∼20%) modified HA significantly promoted binding to CD44 and upregulate chondrogenesis of encapsulated MSCs [71]. The abovementioned synthesis of HA-Nor involved multiple synthetic steps, which were time-consuming and caused undesired contamination. To realize the simple synthesis of HA-Nor, *Xiao* et al. designed a simple and green method to synthesize HA-Nor using HA and carbic anhydride through esterification and further crosslinked HA-Nor with DTT to prepare hydrogels. The hydrogel exhibited controllable gelation properties, swelling, and transmittance and was suitable for cell encapsulation owing to its good cytocompatibility. Notably, the additional carboxyl group in HA-Nor prepared using this method provided a reactive site for further modification [67].

### 2.4 Maleic-modified HA-based hydrogels

In addition to the abovementioned molecules for grafting double C=C bond groups, maleic derivatives are other widely used reagents for modifying polymers with amino or hydroxyl groups [72,73]. *Ren* et al. prepared a hydrogel with a double network structure crosslinked between maleimide-modified HA (HA-Mal) and Gel-MA to support the proliferation and chondrogenesis of BMSCs, where cells were seeded on the surface or inside the hydrogel [74]. *Ren* et al. further evaluated the effect of hydrogels with different mechanical strengths prepared using HA-Mal with different molecular weights (4, 10, and 90 ​kDa) and DTT on the stemness and chondrogenesis of BMSCs. Hydrogels with lower mechanical strength can maintain the stemness properties of BMSCs, whereas hydrogels with higher mechanical strength can promote chondrogenesis of BMSCs by increasing the expression of SOX9 and collagen II [75]. In addition, in contrast to the low methacrylate substitution (<30%) due to the hydrolysis of methacrylic anhydride and sensitivity to pH and temperature, *Zhang* et al. prepared a photocrosslinked porous hydrogel using maleic anhydride-modified HA with a high substitution degree (2.27) of acrylate groups and thiol-terminated PEG (PEG-SH). The designed hydrogel exhibited a rapid gelation time of only 15 ​s, regulatable degradation rate, and good cytocompatibility [76].

## 3 Aldehyde HA-based hydrogels for cartilage tissue engineering

**Fig. 5:**
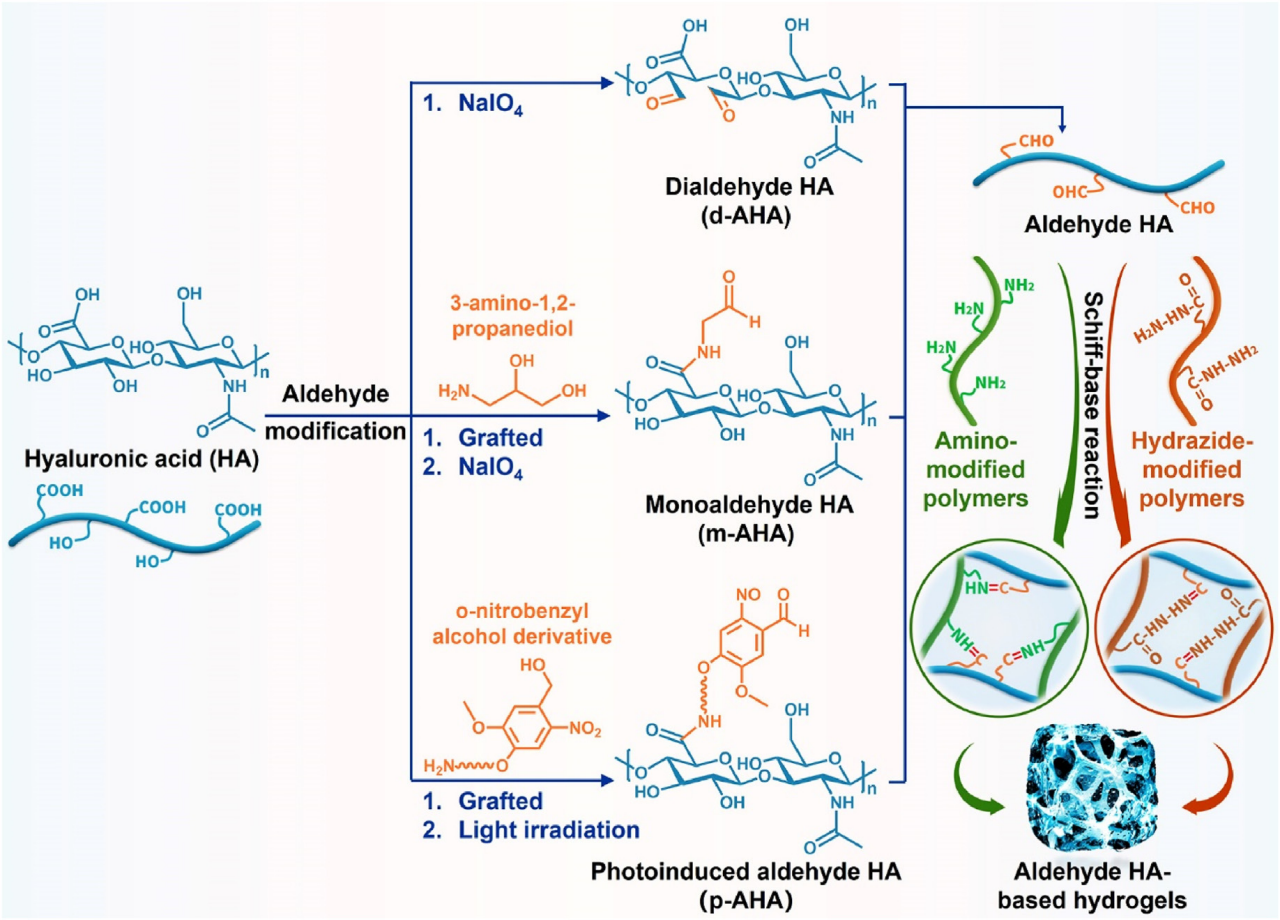
Synthesis of aldehyde HA-based hydrogels for cartilage tissue engineering.

Aldehyde HA-based hydrogels have been widely studied in cartilage tissue engineering because the aldehyde group can undergo a Schiff-base reaction with polymers containing amino and hydrazide groups. The reactions often exhibit the advantages of high speed, simple and mild conditions, and no chemicals. Aldehyde HA can be divided into dialdehyde HA (d-AHA), monoaldehyde HA (m-AHA), and photoinduced aldehyde HA (p-AHA) (Fig. 5 and Table 3).

**Table 3:** Aldehyde HA-based hydrogels for cartilage tissue engineering.

Hydrogels	Components	Physicochemical properties	Biofunctions	Ref.
d-AHA/Chitosan	Dialdehyde HA, chitosan	Controllable stiffness; loading gapmer oligonucleotides	Maintaining chondrocytes spherical morphology and uniform distribution; promoting ECM deposition; inhibiting inflammation	[79,80]
d-AHA/Glycol chitosan	Dialdehyde HA, glycol chitosan, cartilage ECM particles	Suitable compression stress; degradability	Promoting ECM deposition; accelerating cartilage regeneration and integration with surrounding tissues	[81]
d-AHA/water-soluble chitosan	Dialdehyde HA, succinic anhydride-modified chitosan	Tunable degradation rate and mechanical properties	Supporting the adhesion and proliferation of chondrocytes; maintaining the chondrocytes phenotype	[82]
d-AHA/RSV	Dialdehyde HA, amino-functionalized resveratrol	Interconnecting pores	maintaining the chondrocyte phenotype, promoting ECM deposition; reducing inflammation	[84]
d-AHA-MA	Dialdehyde HA, methacrylic anhydride	Higher adhesive strength and adhesion	Accelerating cartilage repair	[85]
m-AHA/Gel-CDH	Monoaldehyde HA, carbohydrazide-modified gelatin	Higher stability; slower degradation rate	Good biocompatibility	[86,87]
m-AHA (aldol crosslinked)	Enolizable monoaldehyde HA, nonenolizable monoaldehyde HA	Fast gelation; excellent hydrolytic stability; good tissue adhesion	Good cytocompatibility	[88]
m-AHA/HA-CDH and HA/CSA·dHCl	Monoaldehyde HA, carbohydrazide-modified HA; HA; cysteamine dihydrochloride	Biphasic, porous and dominant elasticity	Layer-specific inducibility; promoting chondrogenesis of BMSCs and ECM deposition; promoting the osteogenesis of BMSCs	[89]
HA-NB/PRP	HA, *o*-nitrobenzyl alcohol, platelet-rich plasma	Tissue adhesion, controlled release property of growth factor	Accelerating hyaline cartilage regeneration	[90]

d-AHA was synthesized through the oxidation of secondary hydroxyl groups using sodium periodate (NaIO_4_) and then crosslinked with polymers with amino groups using a Schiff-base reaction to engineer hydrogels [77]. In cartilage tissue engineering, chitosan-based hydrogels with many amino groups can provide a suitable microenvironment for chondrocyte adhesion, proliferation, and differentiation [78]. Thus, *Thomas* et al. proved that the stiffness of a hydrogel (HA/chitosan) prepared using d-AHA and chitosan through a Schiff-base reaction had important effects on the growth and functionality of encapsulated chondrocytes. The chondrocytes exhibited a spherical morphology and uniform distribution in the HA/chitosan hydrogel group with less stiffness, whereas better deposition of ECM components, such as GAG and collagen II, was observed in the stiffer hydrogel group [79]. *Cai* et al. further used HA/chitosan hydrogels loaded with gapmer oligonucleotides to inhibit inflammation in OA chondrocytes [80]. Owing to the strong interactions among intermolecular hydrogen bonds, chitosan is almost insoluble in physiological solvents. *Liu* et al. developed a hydrogel with a compression stress of 90 ​kPa at 60% compression strain and with degradable ability using d-AHA, water-soluble glycol chitosan, and cartilage ECM particles incorporated with BMSCs for cartilage repair. The encapsulated BMSCs demonstrated positive activity and proliferation, along with better deposition of GAG and collagen II, which could accelerate cartilage regeneration and integration with surrounding tissues [81]. *Tan* et al. prepared a composite hydrogel with a tunable degradation rate and mechanical properties consisting of d-AHA and water-soluble chitosan modified with succinic anhydride, which could support the adhesion and proliferation of chondrocytes as well as maintain their phenotype [82]. In addition, *Heirani-Tabasi* et al. prepared a chitosan-functionalized HA hydrogel through acylation between carboxyl and amino groups loaded with chondrocyte extracellular vesicles, and the developed hydrogel could upgrade the chondrogenic gene (SOX9 and COL2A1) expression of encapsulated AMSCs and induce the deposition of collagen II and GAG to accelerate hyaline cartilage regeneration in OA cartilage injury [83]. In addition to the aforementioned hydrogels prepared using HA and chitosan, *Sheu* et al. used d-AHA and amino-functionalized resveratrol (RSV) to prepare hydrogels to maintain the chondrocyte phenotype, promote the deposition of cartilage ECM, and reduce lipopolysaccharide-induced inflammation and chondrocyte injury, making them ideal candidates for use as chondrocyte carriers [84]. *Chen* et al. developed a tissue-adhesive one-component hydrogel (d-AHA-MA) that could promote the proliferation and migration of BMSCs. Compared with the adhesive strength of commercial fibrin glue (∼10 ​kPa) and HA-MA hydrogel (∼20 ​kPa), the d-AHA-MA hydrogel exhibited higher adhesive strength (more than 40 ​kPa) and could adhere to native cartilage tissue stably and assist the integration between neo-cartilage and host tissues, facilitating better cartilage regeneration [85].

In addition, unlike d-AHA, m-AHA can be obtained by oxidizing the diol groups grafted onto HA. For example, *Hozumi* et al. used 3-amino-1,2-propanediol to premodify HA, followed by the oxidation of NaIO_4_ to fabricate m-AHA, which was reacted with carbohydrazide (CDH)-modified gelatin (Gel-CDH) to prepare a novel injectable hydrogel (m-AHA/Gel-CDH) through a Schiff-base reaction. Compared with the hydrogels formed from d-AHA and Gel-CDH or adipic dihydrazide (ADH)-modified gelatin (Gel-ADH), the m-AHA/Gel-CDH hydrogel exhibited higher stability and a slower degradation rate because the Schiff-base bond formed by CDH and monoaldehyde was more stable, and the ring-opening structure of polysaccharides was susceptible to hydrolysis. Moreover, the m-AHA/Gel-CDH hydrogel had a pore size of 15–55 ​μm, a shear storage modulus of 0.1–1 ​kPa, and good biocompatibility, which are appropriate for hydrogel scaffolds for soft tissue engineering [86]. *Martinez-Sanz* et al. further demonstrated that a hydrazone-crosslinked hydrogel with insignificant cytotoxicity formed from hydrazide-modified HA and m-AHA was stable under physiological conditions and degradable by hyaluronidase. The hydrogel can controllably release the encapsulated protein and maintain its activity, which has good application prospects in tissue engineering [87]. *Bermejo-Velasco* et al. prepared the first aldol-crosslinked HA-based hydrogel composed of two different *m*-AHAs: an enolizable m-AHA (HA-Eal) and a nonenolizable m-AHA (HA-Nal). The hydrogel had controllable gelation time, excellent hydrolytic stability, hyaluronidase degradability, good tissue adhesion, and good cytocompatibility. Note that the addition of HA-Nal can improve the crosslinking efficiency of the hydrogel owing to the formation of an intramolecular loop in pure HA-Eal hydrogels [88]. Under cartilage tissue engineering, *Chen* et al. prepared an HA-based biphasic hydrogel scaffold using different cross-linking methods to promote osteochondral defect regeneration owing to its layer-specific inducibility. The upper layer of the scaffold with stress relaxation was composed of m-AHA and CDH-modified HA, which can significantly promote the chondrogenesis of BMSCs and deposition of cartilage ECM. The lower layer of the scaffold with porous and dominant elasticity was prepared by retaining the gas generated during the gelation process of HA and cysteamine dihydrochloride, which effectively promoted the osteogenesis of BMSCs. The biphasic hydrogel significantly promoted osteochondral regeneration, similar to native osteochondral tissue *in vivo* [89].

Distinct from the Schiff-base reaction, the photopolymerization reaction is also a typical method for fabricating p-AHA-based hydrogels. For example, *Liu* et al. developed an *in situ* photocrosslinkable composite hydrogel consisting of PRP and *o*-nitrobenzyl alcohol (NB)-modified HA (HA-NB, NB graft ratio: ∼6.0%) that can generate aldehyde groups under light irradiation. The photoinduced crosslinked hydrogel exhibited a tissue adhesive strength of approximately 25 ​kPa and a controlled release of growth factors for hyaline cartilage regeneration [90].

## 4 Thiolated HA-based hydrogels for cartilage tissue engineering

**Fig. 6:**
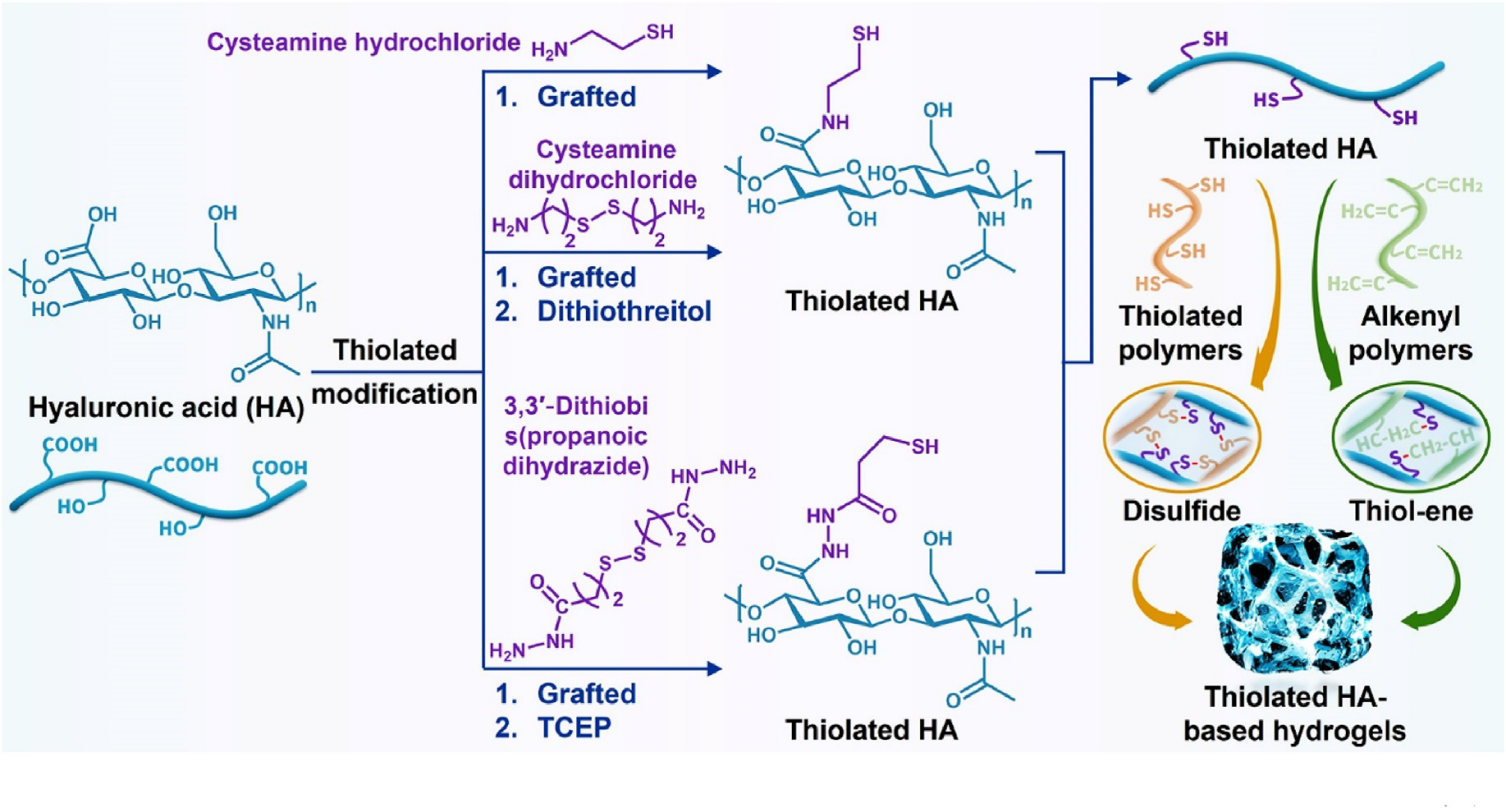
Synthesis of thiolated HA-based hydrogels for cartilage tissue engineering. TCEP: Tris (2-carboxyethyl)phosphine hydrochloride.

Thiolated polymers have a high reactive activity because of their thiol groups and can be further used to prepare hydrogels by self-crosslinking intrachain disulfide bonds or thiol-ene click reactions with rapid gelling time, which have been extensively studied in tissue engineering [91]. Therefore, thiolated HA-based hydrogels have been widely studied for cartilage tissue engineering applications (Fig. 6 and Table 4).

**Table 4:** Thiolated HA-based hydrogels for cartilage tissue engineering.

Hydrogels	Components	Physicochemical properties	Biofunctions	Ref.
HA-SH	HA, cysteamine hydrochloride	Regulatable gelling time, mechanical properties, swelling and degradability	Supporting the culture and delivery of chondrocytes	[92]
HA-SH/peptides	HA, cysteamine hydrochloride, supramolecular peptides	Excellent mechanical properties; abundant cell adhesion sites	Enhancing cartilage-related gene expression and ECM deposition; inhibiting chondrocyte hypertrophy	[93]
HA-SH/TGF-*β*1	HA, 3,3′-dithiobis (propanoic dihydrazide), transforming growth factor-*β*1	Suitable gelation and swelling behavior, sustained release of TGF-*β*1	Promoting chondrogenic gene expression and ECM deposition and distribution	[94,95]
HA-SH/PEG-DVS	HA, cysteamine dihydrochloride, 4-arm polyethylene glycol, divinyl sulfone	Adjustable gelling time; enzymatic degradation	Maintaining the viability and proliferation of chondrocytes; promoting ECM deposition	[96]
HA-SH/HB-PEG	HA, 3,3′-dithiobis (propanoic dihydrazide), hyperbranched polyethylene glycol diacrylate	Regulatable mechanical properties; appropriate degradation rates	Supporting chondrogenic differentiation; promoting hyaline cartilage regeneration	[[97], [98], [99]]
HA-SH/p (HPMAm-lac)-PEG	HA, 3,3′-dithiobis (propanoic dihydrazide), thermosensitive triblock copolymers	Sustained release of HA	Anti-inflammatory ability; inducing chondrogenic differentiation of BMSCs and neo-cartilage formation	[100,101]
HA-SH/Col	HA, cysteamine hydrochloride, collagen I	Improving the adhesion of cells	Maintaining chondrocytes phenotype; promoting ECM deposition	[103]
HA-SH/Col/BCP	HA, cysteamine hydrochloride, collagen I, biphasic calcium phosphate ceramics	Double-layer structure to mimic the condylar osteochondral	Enhancing the regeneration of fibrocartilage and subchondral bone	[104]
HA-SH/Col/icariin-SH	HA, cysteamine hydrochloride, collagen I, thiolated icariin	Controlled-release of bioactive molecules	Inhibiting chondrocyte hypertrophy and dedifferentiation	[105]
HA-SH/Col/HA-Mal	HA, cysteamine hydrochloride, collagen I, maleimide	Biomimetic injectable hydrogel; double-crosslinked network	Promoting the adhesion and proliferation of chondrocytes, GAG deposition and hyaline cartilage formation	[106]
HA-SH/Gel	HA, cysteamine hydrochloride, gelatin	Different bonding intensities	Promoting chondrocyte proliferation; maintenance of the hyaline cartilage phenotype; (strong bonding strength)	[107]

*Bian* et al. fabricated an injectable thiolated HA (HA-SH) hydrogel with regulatable gelling time, mechanical properties, swelling, and degradability through self-crosslinking, which could serve as a 3D scaffold for the culture and delivery of chondrocytes [92]. *Wang* et al. developed a bionic composite hydrogel based on HA-SH and supramolecular peptides using noncovalent supramolecular self-assembly, and the self-assembled hydrogel exhibited excellent mechanical properties and abundant cell adhesion sites. These peptides had beneficial effects on hyaline cartilage formation and phenotype maintenance by promoting cell adhesion and proliferation, enhancing cartilage-related gene expression and ECM deposition, and inhibiting chondrocyte hypertrophy [93]. In contrast to noncovalent crosslinking, *Böck* et al. and *Hauptstein* et al. introduced TGF-*β*1 into HA-SH based hydrogels using covalent bonds *via* Traut's reagent. Better chondrogenesis of BMSCs was verified by promoting chondrogenic gene expression and ECM deposition and distribution [94,95].

Hydrogels prepared based on double C=C bond-functionalized PEG and HA-SH *via* the thiol-ene Michael addition reaction have been widely studied in cartilage tissue engineering. For example, *Jin* et al. prepared an injectable hydrogel with an adjustable gelling time (less than ∼5 ​min) and enzymatic degradation consisting of HA-SH and 4-arm PEG-DVS, which could be advantageous for the viability and proliferation of bovine chondrocytes and deposition of GAG and collagen II [96]. *Li* et al. fabricated a rapid *in situ* crosslinkable hydrogel (HA-SH/HB-PEG) comprising HA-SH and hyperbranched PEG diacrylate with encapsulated arthroscopic flushing fluid-derived MSCs. The obtained hydrogel can support chondrogenesis and hyaline cartilage regeneration in full-thickness cartilage injuries in rats [97]. In addition, the HA-SH/HB-PEG hydrogel can serve as a cellular carrier with an anti-inflammatory ability to aid the viability and proliferation of cartilage-derived progenitor cells (CPCs), together with ECM deposition [98]. *Wang* et al. designed an injectable drug delivery system based on an HA-SH/HB-PEG hydrogel loaded with RSV-containing PLGA microspheres, which exhibited regulatable mechanical properties and appropriate degradation rates for ECM deposition. The delivery system quickly filled cartilage defects *in vivo* for hyaline cartilage regeneration through the controlled and sustained release of RSV [99]. *Sabbieti* et al. and *Agas* et al. prepared a hybrid hydrogel (HA-p (HPMAm-lac)-PEG) according to HA-SH and thermosensitive triblock copolymers (p (HPMAm-lac)-PEG) (composed of poly (*N*-(2-hydroxypropyl) methacrylamide lactate) and PEG) by thermal gelation and Michael addition. The hybrid hydrogel can be used as a carrier for cells and proteins and exhibits good biocompatibility and anti-inflammatory ability through the sustained release of HA. In addition, the HA-p (HPMAm-lac)-PEG hydrogel can also induce chondrogenesis of BMSCs and neo-cartilage formation [100,101].

Collagen I has been extensively used in cartilage tissue engineering owing to its high biocompatibility, low immunogenicity, biodegradability, and availability. However, its poor mechanical properties and rapid degradation rate cannot be ignored [102]. To resolve these limitations, *Chen* et al. fabricated an injectable hybrid hydrogel (HA-SH/Col) by combining collagen I with an HA-SH hydrogel to alleviate the contraction of collagen I and improve the adhesion of cells on the HA-SH hydrogel. The HA-SH/Col hydrogel can be useful for the proliferation and phenotype of chondrocytes as well as the deposition of ECM [103]. *Wang* et al. prepared a double-layer hybrid scaffold based on HA-SH/Col hydrogel and biphasic calcium phosphate (BCP) ceramics to mimic the structure of rabbit condylar osteochondral bone. In a condylar osteochondral injury model, the scaffold enhanced the regeneration of fibrocartilage and subchondral bone with a similar structure and thickness to host cartilage and seamless integration by loading BMSCs and chondrocytes on the upper and lower layers, respectively [104]. *Liu* et al. developed an injectable thiolated icariin-functionalized HA-SH/Col hydrogel for chondrocyte proliferation and cartilage ECM secretion and integration while inhibiting chondrocyte hypertrophy and dedifferentiation [105]. *Yao* et al. prepared a biomimetic injectable hydrogel scaffold combined with collagen I and a double-crosslinked network hydrogel (self-crosslinking of HA-SH and click-crosslinking between HA-SH and HA-Mal) to simulate cartilage ECM and promote the adhesion and proliferation of chondrocytes, GAG deposition, and hyaline cartilage formation [106]. Based on HA-SH and gelatin derivatives, *Wang* et al. prepared three composite hydrogels with different bonding intensities, including physical mixing, weak, and strong bonds. They further evaluated the influence of bonding strength on chondrogenic phenotypes. Compared with hydrogels with physical mixing and weak bonding strength, hydrogels with strong bonding strength exhibit better physical properties, such as a uniform pore structure, water retention performance, and anti-degradation ability. As a result, they can promote chondrocyte proliferation and phenotypic maintenance of hyaline cartilage [107].

## 5 Phenolized HA-based hydrogels for cartilage tissue engineering

**Fig. 7:**
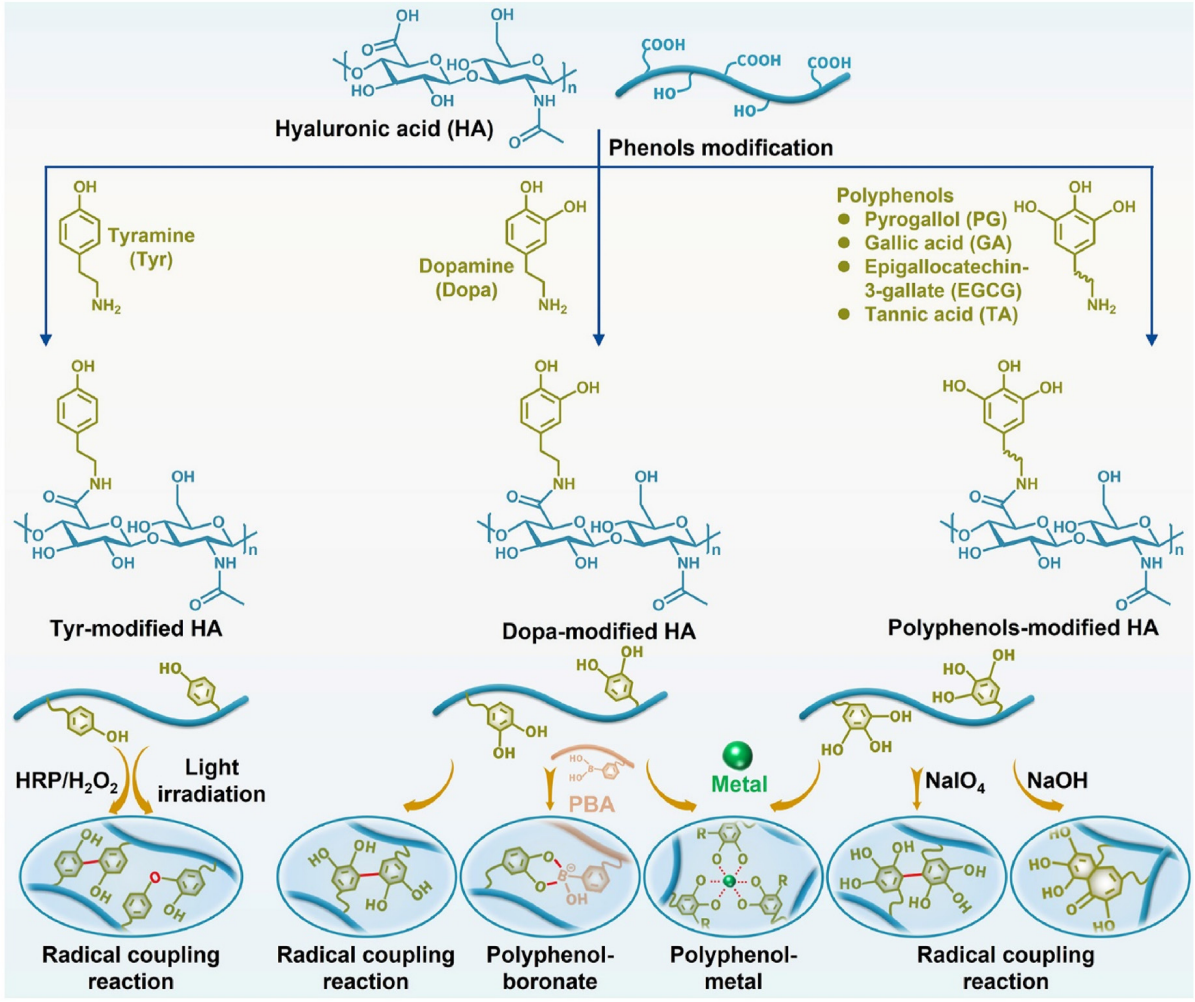
Synthesis of phenolized HA-based hydrogels for cartilage tissue engineering.

Polyphenols are widely found in nature and exhibit good biocompatibility, bioadhesion, and antioxidant properties. Owing to their unique polyphenolic structure, polyphenols can bind other molecules through covalent interactions (Michael addition, Schiff-base reaction, radical coupling reaction, and coordination interactions) and noncovalent interactions (hydrogen bonds and electrostatic and π–π interactions). Therefore, research on polyphenol-modified biomaterials, such as dopamine (Dopa), pyrogallol (PG), gallic acid (GA), epigallocatechin-3-gallate (EGCG), and tannic acid (TA), has gained wide interest [108]. Notably, tyramine (Tyr), a phenolic chemical, also undergoes a radical coupling reaction. Therefore, phenolized HA-based hydrogels have been widely studied in cartilage tissue engineering (Fig. 7 and Table 5).

**Table 5:** Phenolied HA-based hydrogels for cartilage tissue engineering.

Hydrogels	Components	Physicochemical properties	Biofunctions	Ref.
HA-Tyr	HA, tyramine	Controllable crosslinking degree; bioadhesive; delivering cells and bioactive molecules	Promoting formation of hyaline cartilage (lower crosslinked) and fibrocartilage (higher crosslinked); anti-inflammatory ability; enhancing ECM deposition; accelerating cartilage repair	[[109], [110], [111], [112], [113]]
HA-Tyr/SF	HA, tyramine, silk fibroin	Adjustable mechanical properties; sustained release of the drug	Promoting chondrogenic marker genes expression and ECM deposition	[116,118]
HA-Tyr/Gel-Tyr	HA, tyramine, gelatin	Suitable mechanical properties; electrical conductivity	Enhancing the chondrogenic differentiation of BMSCs under electrical stimulation	[119]
HA-Dopa	HA, dopamine	Enhanced adhesion	Reducing cartilage friction and wear to protect cartilage	[126]
d-AHA-Dopa	Dialdehyde HA, dopamine	Higher tissue bonding strength than HA-Dopa; rapid gel formation	Good cytocompatibility	[127]
HA-MA-Dopa	HA, methacrylic anhydride, dopamine	Tissue adhesion; enhanced cell-tissue interaction	Promoting chondrogenesis of hMSCs and cartilage-like matrix formation	[128,129]
HA-MA-Dopa/Fe^3+^	HA, methacrylic anhydride, dopamine, Fe^3+^	Increasing mechanical strength and rapid self-healing performance	As a soft and tough scaffold in the replacement of biological tissues	[130]
HA-furan-Dopa/HA-furan-PBA	HA, furfurylamine, dopamine, phenylboronic acid	pH responsiveness; adhesion; anti-degradation and superior mechanical properties	Maintaining the viability and proliferation of cells; reducing the cell death caused by dopamine oxidation	[131]
HA/Dopa/Col	HA, dopamine, collagen	ECM-mimicking; double crosslinked network	Inhibiting inflammation, promoting full-thickness cartilage repair	[132]
HA-PG	HA, pyrogallol	Different gelation kinetics, mechanical properties, degradability and tissue adhesion	Good biocompatibility	[133]
HA-GA	HA, gallic acid	Tissue-adhesive; enhanced stability; antioxidant	Inhibiting inflammation	[134]
HA-EGCG	HA, ethylamine-grafted epigallocatechin-3-gallate	Resistance to hyaluronidase-mediated degradation; scavenging of free radicals	Inhibiting inflammation; protecting cartilage	[135,136]

### 5.1 Tyramine-modified HA-based hydrogels for cartilage tissue engineering

Tyr-modified HA (HA-Tyr) is synthesized using the acylation reaction between the carboxyl group of HA and the amine group of Tyr, which can be used to prepare HA-Tyr-based hydrogels through catalytic reactions with either hydrogen peroxide (H_2_O_2_) and horseradish peroxidase (HRP) or vitamin B2 derivatives and visible-light-induced photocrosslinking [109,110]. Subsequently, *Toh* et al. indicated that a less crosslinked HA-Tyr-based hydrogel was beneficial to hyaline cartilage formation by enhancing the aggregation and chondrocyte phenotype of BMSCs and increasing the deposition of GAG and collagen II. In contrast, the highly crosslinked HA-Tyr-based hydrogel facilitated the fibrous phenotype of BMSCs and fibrocartilage formation [111]. *Behrendt* et al. suggested that bioadhesive HA-Tyr-based hydrogels can efficiently load and deliver cells to reduce the potential harm caused by cell invasion. The HA-Tyr-based hydrogel maintains its mechanical properties and activates endogenous TGF-*β*1 under joint loading to benefit cartilage repair [112]. Moreover, HA-Tyr-based hydrogels were more favorable for the proliferation and pluripotency of human embryonic stem cells (hESCs) than Tyr-modified dextran hydrogels [113]. However, compared with the HA-Tyr hydrogel, the fibrinogen-HA-based hydrogel had a better effect in promoting endogenous BMSCs migration and cartilage formation. The fibrinogen-HA-based hydrogel can further accelerate the chondrogenesis of endogenous BMSCs by inhibiting the expression of miR-221 [114,115].

In addition to single HA-Tyr-based hydrogels, other natural polymers have been reported to be combined with HA-Tyr for cartilage tissue engineering. As a typical example, *Ziadlou* et al. developed an injectable hybrid hydrogel (HA-Tyr/SF) based on HA-Tyr and silk fibroin (SF) for drug delivery and cartilage tissue engineering. The HA-Tyr/SF hydrogel exhibited adjustable mechanical properties and sustained release of the drug and promoted chondrogenic marker gene expression and ECM deposition [116]. *Wang* et al. further promoted the recruitment of MSCs and cartilage repair using aptamer-functionalized HA-Tyr/SF-based hydrogels [117]. *Weitkamp* et al. developed a highly tunable hybrid hydrogel based on HA-Tyr and SF matrices to maintain the long-term viability of encapsulated chondrocytes and upgrade the expression of chondrogenic marker genes, as well as ECM deposition [118]. In addition, *Vaca-González* et al. prepared an injectable hydrogel with Tyr-functionalized gelatin and HA-Tyr to enhance chondrogenesis of encapsulated BMSCs by promoting the expression of chondrogenic markers and deposition of GAG and collagen II under electrical stimulation [119].

HA-Tyr-based hydrogels have also been extensively investigated for the sustained release of bioactive molecules. For instance, *Kim* et al. prepared a dexamethasone (DMT)-loaded HA-Tyr-based hydrogel to optimize the therapeutic effect on collagen-induced rheumatoid arthritis (RA) by reducing interleukin-6 (IL-6), prostaglandin E2, and the levels of four cytokines, including IL-8, IL-1*β*, tumor necrosis factor-*α* (TNF-*α*), and CD 14 [120]. *Jooybar* et al. utilized platelet lysate (PL)-loaded HA-Tyr-based hydrogels to promote the adhesion and spreading of BMSCs and enhance the deposition of cartilage-like ECM, including collagen II and proteoglycans. Notably, the simultaneous degradation of the hydrogel and deposition of the ECM facilitated the formation of a dense and tough matrix [121]. *Ren* et al. prepared a thermally triggered injectable hydrogel comprising HA-Tyr, HRP-encapsulated thermoresponsive liposomes, and H_2_O_2_ to achieve gelation *in vivo* through the release of HRP because of the phase transition behavior of the liposomes at body temperature. The hydrogel exhibited a controlled gelation rate and desirable activity of encapsulated chondrocytes to support the deposition of GAG and collagen II [122,123].

### 5.2 Polyphenol-modified HA-based hydrogels for cartilage tissue engineering

As a mussel biomimetic material, Dopa can significantly improve tissue adhesion and cell affinity; therefore, it has been widely studied in tissue engineering [124,125]. To improve the adhesion of HA to the surface of articular cartilage, *Ren* et al. developed Dopa-conjugated HA (HA-Dopa) using Dopa and HA through an acylation reaction. Compared with HA, HA-Dopa can effectively adhere to the cartilage surface and enhance boundary lubrication, which can reduce cartilage friction and wear to protect cartilage [126]. However, the HA-Dopa hydrogel suffered from insufficient adhesiveness and overfast degradation owing to the low grafting rate of Dopa. To solve these problems, *Zhou* et al. developed a high-tissue-adhering hydrogel (d-AHA-Dopa) using d-AHA and Dopa *via* a Schiff-base reaction. The d-AHA-Dopa hydrogel exhibited a higher tissue bonding strength (∼90 ​kPa) than the HA-Dopa hydrogel (∼10 ​kPa), rapid gel formation (less than 60 ​s), degradability, and good cytocompatibility, making it a promising bioadhesive [127]. *Salzlechner* et al. prepared a Dopa-biofunctionalized HA-MA (HA-MA-Dopa) hydrogel with tissue adhesion at the defect site. As a result, the adhesive hydrogel presented enhanced cell-tissue interaction owing to the presence of the Dopa group, and it had significant positive influences on the chondrogenesis of hMSCs and the formation of the cartilage-like matrix [128,129]. Notably, compared with single-crosslinked hydrogels, double-crosslinked hydrogels exhibit better mechanical properties, stability, and antidegradability. *Guo* et al. further demonstrated a dynamic reversible double-crosslinked network hydrogel through the catechol of HA-MA-Dopa and Fe^3+^ and photopolymerization to increase its mechanical strength and rapid self-healing performance, and biological studies further indicated promising application prospects of the obtained hydrogel as a soft and tough scaffold for the replacement of biological tissues such as cartilage [130]. *Yu* et al. prepared a dual-crosslinked HA-based hydrogel using Dopa/furfurylamine (furan)-modified HA (HA-furan-Dopa) and phenylboronic acid (PBA)/furan-modified HA (HA-furan-PBA) *via* a Diels–Alder click reaction and phenylboronic ester bond. The hydrogel exhibited injectability, pH responsiveness, adhesion, anti-degradation, and superior mechanical properties. In addition, the hydrogel can maintain the viability and proliferation of encapsulated cells, and the introduced PBA group can reduce cell death caused by Dopa oxidation, which exhibits good application prospects in cartilage tissue engineering [131]. *Gan* et al. prepared an ECM-mimicking hydrogel using Dopa noncovalently modified HA (HA/Dopa) and collagen with a double-crosslinked network for growth factor-free cartilage regeneration. The hydrogel exhibited a better affinity for cells, inhibition of the expression of inflammatory factors, and M2 polarization of macrophages, which significantly promoted full-thickness cartilage repair of a rabbit knee [132].

In addition to Dopa, other polyphenols have been extensively studied for the functional modification of HA. Similar to the synthesis of HA-Dopa, *Cho* et al. prepared a fast-forming hydrogel (HA-PG) using HA and PG through dual modes of oxidative crosslinking, including oxidant and pH control. Based on the different crosslinking modes, the HA-PG hydrogels exhibited different gelation kinetics, mechanical properties, degradability, and tissue adhesion. Furthermore, both HA-PG hydrogels exhibited good biocompatibility, providing multifunctional hydrogels for tissue engineering, drug delivery, and stem cell therapy [133]. GA, a small molecule polyphenol, has various biological activities, such as anticarcinogenic, antimutagenic, and anti-inflammatory properties. *Samanta* et al. designed a tissue-adhesive antioxidant HA-based hydrogel (HA-GA) using HA and GA *via* covalent crosslinking. The HA-GA hydrogel exhibited enhanced stability owing to the crosslinked GA network. HA-GA hydrogels can polarize macrophages to an immunosuppressive phenotype and inhibit the expression of pro-inflammatory factors, which has significant potential in inflammation-related biomedical applications [134]. EGCG is a polyphenol in green tea with anticancer and anti-inflammatory activities. *Jin* et al. prepared an injectable hybrid hydrogel based on HA-Tyr and gelatin to load EGCG for cartilage repair in OA patients. The hybrid hydrogel inhibited inflammation and protected the cartilage in surgery-induced OA [135]. *Lee* et al. prepared an HA-EGCG conjugate using HA and ethylamine-grafted EGCG. Compared with HA, the EGCG-HA conjugate exhibited resistance to hyaluronidase-mediated degradation and scavenging of free radicals, which has good application prospects in arthritis treatment [136]. TA is a large molecular polyphenol with various intrinsic properties, such as antioxidant, metal chelation, and polymerization properties. *Gwak* et al. and *Lee* et al. demonstrated that the addition of TA can enhance the adhesion, compression properties, and biostability of HA-based hydrogels owing to strong hydrogen bonding and covalent crosslinking [137,138].

**Fig. 8:**
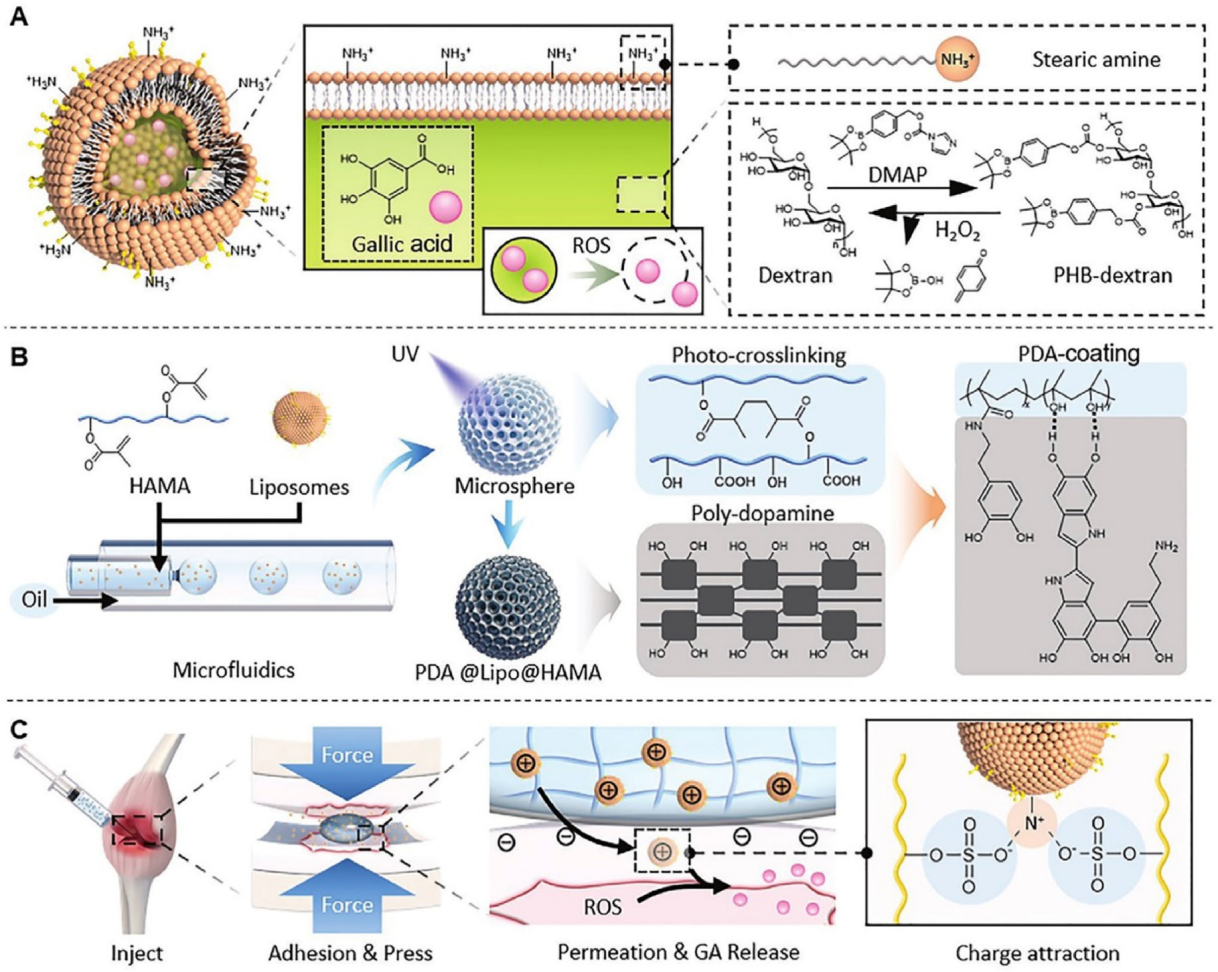
Schematic illustration of the fabrication of charge-guided nanohydrogel microsphere to penetrate the cartilage matrix. (A) Preparation of positive charges liposomes; (B) preparation of PDA@Lipo@HAMA nanohydrogel microsphere; (C) design of charge-guided microsphere for cartilage tissue engineering [139]; Copyright 2021, John Wiley and Sons.

Because the cartilage ECM has a dense structure and a high density of negative charges, the delivery of drugs into cartilage is a challenge. To address this problem, *Lin* et al. fabricated charge-guided nanohydrogel microspheres through HA-MA, Dopa, and liposomes using microfluidic technology to penetrate the cartilage matrix. The microspheres were surface-modified with Dopa to optimize their adhesion to the cartilage surface. Moreover, the positively charged liposomes prepared from stearyl amine and borate-dextran deeply penetrated the cartilage ECM and achieved ROS-responsive release of GA on chondrocytes. In addition, in a rat OA model, microspheres significantly alleviated OA progression by enhancing the infiltration of cartilage ECM, antioxidant efficacy, and inhibiting oxidative stress-induced chondrocyte apoptosis (Fig. 8) [139].

## 6 Hydrazide HA-based hydrogels for cartilage tissue engineering

**Fig. 9:**
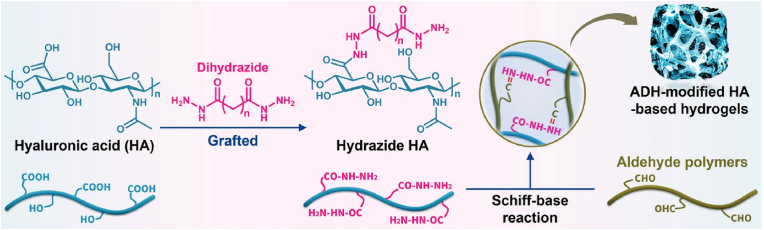
Synthesis of hydrazide HA-based hydrogels for cartilage tissue engineering.

Polymers can be modified by hydrazide to endow them with abundant hydrazide groups [140]. Among the various hydrazide compounds, ADH is commonly used to modify HA, and ADH-modified HA-based hydrogels have been widely studied in cartilage tissue engineering (Fig. 9 and Table 6).

**Table 6:** Hydrazide HA and host-guest group-modified HA-based hydrogels for cartilage tissue engineering.

Hydrogels	Components	Physicochemical properties	Biofunctions	Ref.
HA-ADH/d-AHA	HA, adipic dihydrazide, dialdehyde HA	Shear-responsive; boundary lubricated; anti-inflammatory; injectable; self-healing; controlled-release of bioactive molecules	Reducing friction; alleviating the progression of OA; inhibiting inflammation; protecting chondrocytes	[141,142]
HA-ADH/dextran	HA, adipic dihydrazide, dialdehyde dextran	ROS depletion, sustainable drug release; viscosupplementation	Inhibiting inflammation; alleviating the progression of OA; protecting cartilage	[143]
HA-ADH/pectin	HA, adipic dihydrazide, oligopeptide-grafted dialdehyde pectin	Short gel times; controllable mechanical properties and degradation capacity	Providing a suitable microenvironment for the phenotype of chondrocytes and chondrogenesis	[144]
HA-Fur/CS-Mal/ACS/ADH	Methylfuran-modified HA, maleimide-modified chondroitin sulfate, adipic dihydrazide, dialdehyde chondroitin sulfate	Double-network; controllable viscoelasticity and stress relaxation; good processability; self-healing ability; high swelling capacity and stability	Negligible inflammatory response	[145]
HA-Ad/*β*-CD-Ac	HA, adamantane, monoacrylated *β*-cyclodextrin	Good self-healing properties, compressibility; sustained release of TGF-*β*1	Rapidly integrating with the defect tissue; promoting the chondrogenesis of MSCs and cartilage regeneration	[146]
HA-Ad/*β*-CD-SH/HA-Mal	HA, adamantane, thiolated *β*-cyclodextrin, maleimide	Tunable viscoelasticity	Maintaining 3D spreading and intracellular interactions of encapsulated MSCs	[147]
HA-*β*-CD/PAA-Fer	HA, *β*-cyclodextrin, ferrocene, polyacrylic acid	Magnetic navigation; controlled release of glutathione	Promoting the columnar arrangement of chondrocytes and the repair of cartilage damage	[148]
HA-CB [6]/HA-PA	HA, cucurbit [6]uril, polyamines	Good mechanical stability; enzymatic degradability	Biocompatibility	[149]

*Lei* et al. developed a shear-responsive, boundary-lubricated, anti-inflammatory, injectable, and self-healing HA-based hydrogel (HA-ADH/d-AHA) using ADH-modified HA (HA-ADH) and d-AHA through a Schiff-base reaction to deliver celecoxib (CLX)-loaded liposomes. The hydrogel can be restructured to expose the internal liposome on the outer surface under shear induction and form a stable lubricated boundary layer to reduce friction while alleviating the progression of OA by releasing CLX [141]. *Zhou* et al. further used an HA-ADH/d-AHA hydrogel to load bovine serum albumin-modified dioxide manganese nanoparticles and PRP. Biological experiments have indicated that the hydrogel can significantly increase the proliferation of chondrocytes and alleviate OA *in vivo* by inhibiting inflammation and protecting chondrocytes from oxidative stress [142]. *Zhou* et al. prepared a novel multifunctional complex hydrogel with ROS depletion, sustainable drug release, and viscosupplementation for combinatorial therapy of osteoarthritis. The complex hydrogel was fabricated using HA-ADH and aldehyde dextran through a Schiff base reaction, which can achieve the loading and sustained release of dexamethasone acetate-encapsulated ROS-scavenging micelles (PDMs). The HDH@PDM hydrogels exhibited good biodegradability, similarity to synovial components, and antioxidant and anti-inflammatory properties. Furthermore, in a rat OA model, the HDH@PDM hydrogel inhibited inflammation by polarizing macrophages to the anti-inflammatory M2 phenotype to alleviate OA progression and protect cartilage [143]. *Chen* et al. used HA-ADH and oligopeptide-grafted dialdehyde pectin to prepare an injectable hydrogel with short gel times (112–399 ​s), controllable mechanical properties, and degradation capacity, which provided a suitable microenvironment for chondrogenesis and chondrogenesis [144]. In addition, *Mihajlovic* et al. prepared a double-network hydrogel based on Diels–Alder adducts between methylfuran-modified HA (HA-Fur) and maleimide-modified CS (CS-Mal), and hydrazone bonds between ADH and dialdehyde CS. The hydrogel exhibited controllable viscoelasticity and stress relaxation, good processability and self-healing ability, and a high swelling capacity and stability. In addition, the hydrogel had mechanical properties similar to those of cartilage tissue to support the activity and proliferation of BMSCs. Interestingly, the degradation products of the manufactured hydrogel did not cause an inflammatory response in macrophages, laying the foundation for its further application in cartilage tissue engineering [145].

## 7 Host–guest group-modified HA-based hydrogels for cartilage tissue engineering

**Fig. 10:**
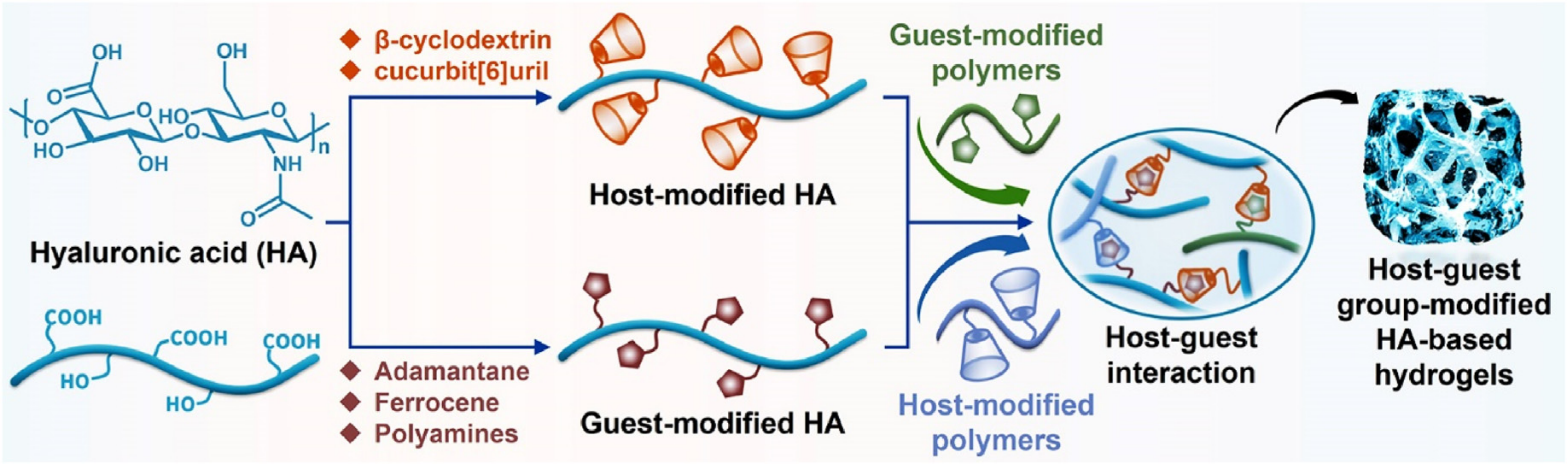
Synthesis of host–guest-modified HA-based hydrogels for cartilage tissue engineering.

In addition to the abovementioned methods for preparing HA-based hydrogels *via* covalent bonding, noncovalent crosslinking, such as host–guest interactions, has also been investigated to create HA-based hydrogels for cartilage tissue engineering (Fig. 10 and Table 6).

As a typical example, *Wei* et al. prepared a supramolecular hydrogel based on adamantane-functionalized HA (HA-Ad) and monoacrylated *β*-cyclodextrin (*β*-CD-Ac) through host–guest interactions and radical polymerization. The hydrogel exhibited excellent self-healing properties, compressibility, and sustained release of TGF-*β*1. Moreover, the hydrogel could rapidly integrate with the defective tissue and promote chondrogenesis of MSCs and cartilage regeneration *in vivo* [146]. *Jing* et al. prepared a highly dynamic network hydrogel with tunable viscoelasticity using HA-Ad, HA-Mal, and thiolated *β*-CD through host–guest interactions and thiol-ene click reactions. The hydrogel can maintain the 3D spreading and intracellular interactions of encapsulated MSCs [147]. *Chiang* et al. first developed a smart injectable composite hydrogel using *β*-CD-modified HA (HA-*β*-CD) and ferrocene-modified polyacrylic acid through host–guest interactions to encapsulate glutathione (GSH)-loaded magnetic microcapsules. The hydrogel exhibited magnetic navigation and controlled release of GSH, which can effectively promote the columnar arrangement of chondrocytes and the repair of cartilage damage [148]. *Park* et al. prepared a biocompatible hydrogel using cucurbit [6]uril (CB [6])-conjugated HA and polyamine (1,6-diaminohexane or spermine)-conjugated HA, which achieved *in situ* gelation through host–guest interaction after subcutaneous injection into nude mice. The hydrogels exhibited excellent mechanical stability, enzymatic degradability, and biocompatibility, and they have application potential in cell culture and various tissue engineering applications [149].

## 8 HA-based 3D bioprinted hydrogels for cartilage tissue engineering

Articular cartilage is lubricated lamellar tissue with anisotropy and heterogeneity [150]. Artificial cartilage scaffolds prepared using traditional engineering methods have difficulty precisely mimicking the biomechanics and tissue composition of natural cartilage [151]. On one hand, a novel 3D bioprinting method can control the geometry and microstructure of hydrogel scaffolds, which has a prosperous application in regulating the mechanical properties of hydrogel scaffolds and maintaining the viability and function of loaded cells, drugs or cytokines [152,153]. On the other hand, the HA solution has an ideal viscous modulus for 3D printing, but it cannot be used as a bioink for 3D printing alone because of the lack of specific shape-retaining ability. Therefore, HA has been integrated with other components to create bioinks for 3D printing, and HA-based bioinks can be divided into hybrid and chemically modified bioinks [154] (Table 7).

**Table 7:** HA-based 3D bioprinted hydrogels for cartilage tissue engineering.

3D bioprinted hydrogels	Components	Physicochemical properties	Biofunctions	Ref.
HA/PU	HA, polyurethane	High elastic recovery; processability	Promoting self-aggregation and chondrogenesis of MSCs	[155]
HA/alginate	HA, alginate	Printability; biodegradability	Promoting chondrogenic genes expression and ECM deposition	[157]
HA/elastin/Gel	HA, elastin, gelatin	Printability with reproducible outcomes	Supporting the penetration, proliferation and chondrogenesis of chondrocytes	[158]
HA-MA/Gel-MA	HA, gelatin, methacrylic anhydride (MA)	Controllable pore structure; optimum degradation rate	Maintaining chondrocyte phenotype, promoting ECM deposition; accelerating mature cartilage regeneration	[[160], [161], [162]]
HA-MA/PCL	HA, MA, polycaprolactone	Biomimetic multiphase structure; sustained release of drugs	Inhibiting inflammation, repairing osteochondral damage; restoring motor function of joints	[163]
HA-MA/ECM	HA, MA, ECM	Improving the compressive strength and modulus	Providing a suitable microenvironment to encapsulate and cultivate cells	[164]
HA-MA/p (HPMAm-lac)-PEG	HA, MA, triblock copolymers	Thermosensitive, improving printability	Promoting formation of hyaline cartilage (lower concentrations) and fibrocartilage (higher concentrations)	[165]
HA-MA-PBA/Gel-MA/HA-Dopa	HA, MA, phenylboronic acid, gelatin, dopamine	Microporosity; injectability; tissue adhesion; sustained release of drug	Promoting chondrogenesis of BMSCs; accelerating articular cartilage repair and regeneration	[166]
HA-Ac-PBA/PVA/Gel-HS	Acrylated HA, phenylboronic acid, polyvinyl alcohol, thiolated gelatin	Tunable viscoelasticity; shear thinning property; high structural fidelity	Maintaining chondrocyte phenotype; promoting chondrogenesis and ECM deposition; alleviating chondrocyte damage	[167]
HA-GM/Gel-MA	HA, glycidyl methacrylate, gelatin, MA	Excellent stable mechanical properties and printability	Good biocompatibility; providing a suitable microenvironment for the chondrogenesis	[168]
HA-SH/P (AGE-co-G)	Thiolated HA, allyl-modified polyglycidol	Improving the stiffness of hydrogel	Promoting the deposition and uniform distribution of ECM	[169,170]
hmHA-HS/PEG	Thiolated high molecular weight HA, acrylated PEG, allylated PEG	Better stiffness of the hydrogel	Promoting homogeneous distribution of ECM	[171]
HA-SH/ECM	Thiolated HA, ECM particles	Customizable specific shapes; biomimetic mechanical properties	Enhancing the viability of the encapsulated chondrocytes	[173]
HA-ADH/SAV and SA/Ca^2+^ (HBSAC)	biotinylated HA-ADH, streptavidin, sodium alginate, Ca^2+^	Better printability and structural integrity	Promoting the chondrogenic marker genes expression and ECM deposition	[174]
d-AHA/glycol chitosan/ADH and HAH/Ca^2+^	Dialdehyde HA, glycol chitosan, ADH, hyaluronate-alginate hybrid, Ca^2+^	improving the mechanical properties and stability	Enhancing the chondrogenesis of chondroprogenitor cells	[175]
HA-ADH/d-AHA/Gel-MA	HA-ADH, dialdehyde HA, gelatin, MA	Self-healing; shear-thinning properties; improving mechanical properties	Maintaining the survival and proliferation of BMSCs	[176]
HA-Tys	HA, tyramine	Controllable porosity; enhanced the mechanical strength;	Loading and delivering stem cells and chondrocytes with appropriate cell activity for a long time	[177,178]
d-AHA/CMC and Gel/PEG-SG	Dialdehyde HA, *N*-carboxymethyl chitosan, gelatin, PEG succinimidyl glutarate	Viscoelastic; time-sharing structure-supporting; high permeability	Preserving the long-term viability and morphology of chondrocytes	[179]

### 8.1 3D bioprinted hydrogels based on hybrid HA

*Hung* et al. prepared a 3D printed scaffold based on HA and water-based polyurethane (PU) elastic nanoparticles to control the release of bioactive ingredients or drugs for customized cartilage tissue engineering. The printed scaffold had a high elastic recovery ability and processability for various shapes, which boosted the self-aggregation and chondrogenesis of MSCs while inhibiting cartilage hypertrophy. In addition, cartilage regeneration in rabbit knee-joint defects was obtained [155]. In addition, *Shie* et al. used HA and light-cured PU to prepare a 3D printed scaffold mimicking the mechanical properties of articular cartilage. The prepared scaffold was favorable for the adhesion, proliferation, and chondrogenesis of MSCs [156]. *Antich* et al. illustrated a biomimetic 3D printable bioink based on HA and alginate, and chondrogenesis of chondrocytes was observed by promoting chondrogenic gene expression and cartilage ECM deposition [157]. *Shokri* et al. designed a porous 3D printed scaffold composed of HA, elastin, and gelatin, which supported the penetration, proliferation, and chondrogenesis of chondrocytes, along with the regeneration of nasal septal cartilage defects [158].

### 8.2 3D bioprinted hydrogels based on functionally modified HA

**Fig. 11:**
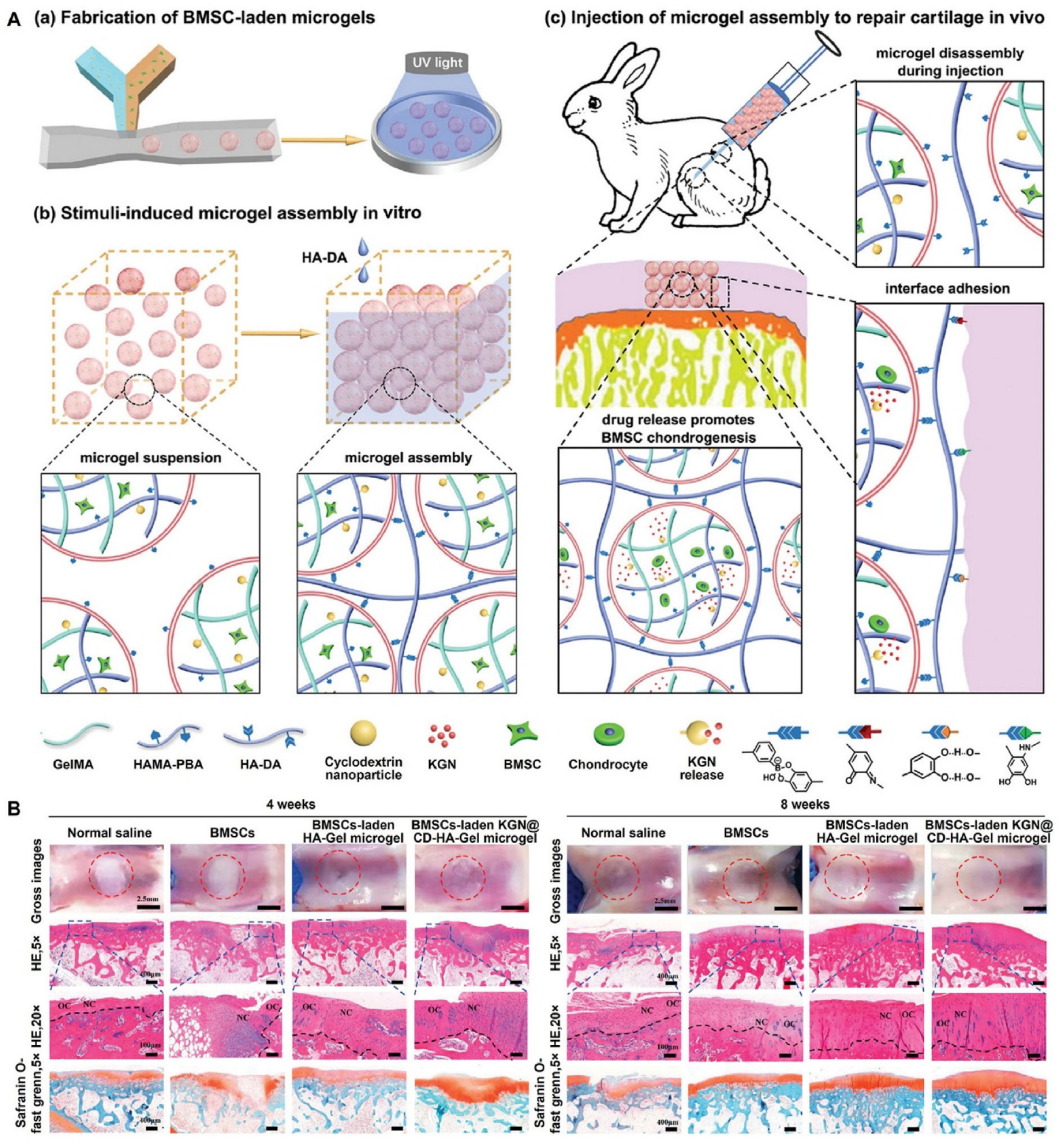
Schematic illustration of the fabrication of dynamic-crosslinked microgel for cartilage regeneration. (A) Microgel with microporosity, injectability, tissue-adhesion, and sustained drug release; (B) immunohistochemical staining images of cartilage defects in different groups after implantation for 4 and 8 weeks [166]; Copyright 2021, John Wiley and Sons.

Functionalized HA-based hydrogels have application prospects in cartilage tissue engineering [19]. Similarly, they are also elaborately designed and 3D printed to biomimetic the structure and shape of native cartilage [159]. *Lam* et al. developed two 3D bioprinted hydrogels consisting of HA-MA and Gel-MA using stereolithographic methods to maintain the chondrocyte phenotype and promote the deposition of cartilage-specific ECM, including proteoglycans and collagen II [160]. *Xia* et al. used HA-MA and Gel-MA to prepare a 3D bioprinted hydrogel by integrating photocuring and lyophilization techniques. The controllable pore structure, desirable mechanical properties, and optimum degradation rate matched those of native cartilage regeneration. In addition, hydrogels loaded with chondrocytes can contribute to mature cartilage regeneration with typical lacunar structures and cartilage-specific ECM [161]. *Shopperly* et al. also developed a biomimetic 3D bioprinted hydrogel with a structure similar to that of cartilage ECM gradients based on HA-MA and Gel-MA *via* stereolithographic bioprinting. The increased HA-MA and Gel-MA contents enhanced the stiffness of the scaffolds and cartilage matrix protein formation. In addition, the biomimetic structure of the 3D printed hydrogel could be retained even after ECM formation [162]. *Liu* et al. developed a biomimetic multiphase composite 3D bioprinted hydrogel combining HA-MA with PCL to simulate the osteochondral structure. The bioprinted hydrogel supported the survival and proliferation of BMSCs and deposition of cartilage ECM. In addition, the hydrogel could inhibit inflammation, repair osteochondral damage, and restore the motor function of OA joints after loading with small-molecule drugs [163]. *Li* et al. prepared an ECM-doped HA-MA-based 3D bioprinted hydrogel to improve the compressive strength (2.7 times) and modulus (3.1 times) of HA-MA hydrogels. The hydrogels can both satisfy the mechanical requirements of bioprinting and provide a suitable microenvironment for encapsulating and cultivating cells [164]. In addition, *Mouser* et al. designed a thermosensitive 3D bioprinted hydrogel by incorporating triblock copolymers (p (HPMAm-lac)-PEG) into HA-MA to improve its printability for chondrogenesis. Notably, lower concentrations of HA-MA (0.25%–0.5%) had beneficial effects on the deposition of GAG and collagen II, while higher HA-MA concentrations (1%) could induce fibrocartilage formation [165]. *Feng* et al. used PBA-grafted HA-MA (HA-MA-PBA) and Gel-MA to fabricate a microgel using microfluidics and photocrosslinking to encapsulate KGN-loaded cyclodextrin nanoparticles and BMSCs and then prepared a nanocomposite microgel through dynamic crosslinking between PBA groups and HA-Dopa. The microgel exhibited microporosity, injectability, tissue adhesion, and sustained drug-release ability. Moreover, the microgel accelerated chondrogenesis of BMSCs and the repair and regeneration of articular cartilage (Fig. 11) [166]. *Shi* et al. prepared an injectable dynamic double-crosslinked 3D bioprinted hydrogel comprising PBA-grafted acrylated HA (HA-Ac-PBA), polyvinyl alcohol (PVA), and Gel-HS through boronate ester bond and thiol-ene click reactions. The double-crosslinked hydrogels exhibited tunable viscoelasticity, shear thinning, and high structural fidelity. After encapsulating AMSCs, the hydrogel promoted cell adhesion and chondrogenesis, maintained the chondrocyte phenotype, increased the deposition of GAG and collagen II, and alleviated chondrocyte damage from ROS, suggesting decent application prospects of hydrogels in OA cartilage repair [167]. *Lee* et al. fabricated a photocurable 3D bioprinted hydrogel with HA-GM and Gel-MA, which exhibited excellent stable mechanical properties and printability, enabling the construction of highly complex laryngeal structures (thyroid cartilage, cricoid cartilage, arytenoid cartilage, etc.). Moreover, the hydrogel had good biocompatibility and provided a suitable microenvironment for the chondrogenesis of tonsil-derived mesenchymal stem cells [168].

*Stichler* et al. engineered a 3D bioprinted hydrogel (HA-SH/P (AGE-co-G)) based on HA-SH and allyl-modified polyglycidol (P (AGE-co-G)), which could encapsulate MSCs and promote the deposition of ECM, although the distribution of ECM was not uniform [169]. *Hauptstein* et al. further demonstrated that HA-SH/P (AGE-co-G) hydrogel with a low concentration (3%) of HA-SH polymer contributed to the uniform distribution of ECM and significantly improved the stiffness of the hydrogel. In addition, the addition of high-molecular-weight HA (hmHA) further optimized the distribution of ECM and stiffness of the hydrogel [170]. Subsequently, *Hauptstein* et al. fabricated a 3D bioprinting hydrogel with a loose porous structure based on low concentrations of hmHA-HS, acrylated PEG, and allylated PEG through a two-stage cross-linking method. A homogeneous distribution of proteoglycans and collagen II was obtained, and the improved stiffness of the hydrogel after chondrogenesis was also verified [171]. *Mancini* et al. developed a 3D bioprinted zonal composite scaffold combining HA-SH/P (AGE-co-G) with PCL to encapsulate articular cartilage progenitor cells (ACPCs) on the top layer and MSCs on the bottom layer to mimic the zonal structure of the native cartilage. In equine osteochondral defects, significant bone repair and integration with a higher compressive modulus of the repaired tissue was observed on the scaffold with a zonal structure. However, the negligible cartilage repair effect might be related to early loss of cells, undesirable degradation behavior of the hydrogel, and failure to provide the biomechanical environment for native cartilage [172]. *Barthold* et al. fabricated a layered 3D bioprinted hydrogel utilizing HA-SH and ECM particles through disulfide bonds, which exhibited customizable specific shapes and mechanical properties similar to those of the native cartilage. Moreover, the hydrogel enhanced the viability of encapsulated chondrocytes [173].

In addition, HA-ADH-based double-crosslinked network hydrogels have been extensively studied for 3D bioprinting. *Nedunchezian* et al. prepared a double-cross-linked 3D bioprinted hydrogel (HBSAC) to lade adipose stem cells (ADSCs). The double-crosslinked network of the HBSAC hydrogel included noncovalent crosslinks between biotinylated HA-ADH and streptavidin, and ion transfer crosslinks between SA and Ca^2+^. Compared with HA hydrogels, HBSAC hydrogels exhibited better printability and structural integrity and effectively promoted the chondrogenesis of ADSCs by promoting the expression of chondrogenic marker genes and the deposition of GAG [174]. *Kim* et al. prepared an HA-based hydrogel with a double-crosslinked network as 3D printable bioinks. The double-crosslinked network included covalent crosslinks between AHA, glycol chitosan, and ADH, and the physical crosslinks of hyaluronate-alginate hybrid (HAH) polymers with Ca^2+^. The addition of HAH polymers can significantly improve the mechanical properties and stability of hydrogels and enhance chondrogenesis of chondroprogenitor cells [175]. *Wang* et al. prepared a dynamic/photocrosslinked HA-Gel dual-network hybrid hydrogel using HA-ADH, AHA, and Gel-MA for 3D bioprinting. The dynamic crosslinking of HA-ADH and AHA through a Schiff-base reaction endowed the hydrogel with self-healing and shear-thinning properties to ensure its smooth extrusion, and the subsequent photopolymerization of Gel-MA improved its mechanical properties and stability. The hydrogel maintained the survival and proliferation of BMSCs [176].

*Flégeau* et al. reported HA-Tys microgels with controllable porosity for 3D printable bioinks, and the microgels were useful for the activity and chondrogenesis of chondrocytes. Note that the increase in microgel size would induce a more severe inflammatory response [177]. In addition, *Petta* et al. reported a 3D bioprintable HA-Tys hydrogel with a dual crosslinking mechanism. First, enzymatic reaction-induced crosslinking formed a soft gel suitable for cell encapsulation and extrusion. Second, visible light-induced photocrosslinking enhanced the mechanical strength of hydrogels such that they could preserve their shape and morphology. Printed hydrogels can load and deliver stem cells and chondrocytes with appropriate cell activity for a long time [178]. *Chen* et al. demonstrated a self-healing viscoelastic 3D bioprintable hydrogel with novel time-sharing structure-supported characteristics and high permeability through fast dynamic crosslinking between d-AHA and *N*-carboxymethyl chitosan and slow crosslinking between gelatin and 4-arm PEG succinimidyl glutarate. Moreover, hydrogels can preserve the long-term viability, morphology, and remarkable biological characteristics of encapsulated chondrocytes [179].

## 9 Conclusion and further perspectives

HA has been widely applied in cartilage tissue engineering because of its biocompatibility, biodegradability, promotion of cell adhesion and proliferation, regulation of inflammation, and acceleration of cartilage regeneration. It is an important material for the construction of hydrogels/microgels, and functional groups grafted onto HA can serve as cross-linking and reaction sites to further immobilize bioactive factors, thereby improving the bioactivity of HA hydrogels. With the continuous development of science and technology, numerous functionalized HA-based hydrogels have been elaborately created, and they have exhibited good application prospects in cartilage tissue engineering. This article summarizes the research progress in design strategies (alkenyl, aldehyde, thiolated, phenolized, hydrazide, and host–guest group-modified) for HA-based hydrogels and their applications in cartilage tissue engineering.

Among the above functionalization modifications, alkenyl HA has been extensively and thoroughly studied in cartilage tissue engineering. Alkenyl HA, such as methacrylate, DVS-modified, Nor-modified, and maleic-modified HA, is primarily synthesized through esterification and acylation. Methacrylate HA can self-polymerize to form a hydrogel through the FRP reaction; the pore size, crosslinking density, mechanical properties, and degradation rate of the hydrogel can be effectively controlled by regulating the polymer concentration and polymerization time. Moreover, many polymers (such as gelatin, cellulose, fibrinogen, and F127) have been used to further improve the physicochemical properties and biological activities of hydrogels; the process of hydrogel formation does not depend on the functional groups of polymers because of the self-polymerization of methacrylate HA. In contrast, other alkenyl HA molecules are more complex to synthesize, and related hydrogels are typically fabricated through the thiol-ene click reaction with thiolated polymers. Aldehyde HA can be classified into d-AHA, m-AHA, and p-AHA. Aldehyde HA-based hydrogels have been prepared primarily through the Schiff-base reaction with polymers containing amino groups or hydrazide groups; the preparation process is simple and does not require the addition of other chemicals. These hydrogels exhibit excellent tissue adhesion by reacting with the amino group on the tissue surface and self-healing owing to the presence of dynamic Schiff-base bonds. The synthesis of d-AHA requires only one oxidation step, in which NaIO_4_ oxidizes the vicinal hydroxyl groups on the second and third carbon atoms of the HA repeat unit. d-AHA have highly reactive dialdehyde groups that have attracted extensive research interest. In contrast to d-AHA, both m-AHA and p-AHA require a two-step preparation process, including the grafting of functional groups initially followed by oxidation/photoinduction. Interestingly, the ring-opening structure of d-AHA is easily hydrolyzed, leading to rapid degradation and instability of the hydrogel, whereas the hydrogel formed by m-AHA and hydrazide has a higher stability and slower degradation rate because of its more stable Schiff-base bond. Compared with d-AHA and m-AHA, p-AHA can effectively avoid the instability of the aldehyde group. Therefore, m-AHA and p-AHA should be investigated comprehensively in cartilage tissue engineering based on the relatively few studies to date. Thiolated HA is prepared primarily through an acylation reaction between the carboxyl group of HA and the amino or hydrazide groups of polymers with disulfide bonds and sulfhydryl groups. Thiolated HA-based hydrogels have been prepared primarily through self-crosslinking of intrachain disulfide bonds or thiol-ene click reactions. The self-crosslinking process of the hydrogels does not require other chemicals, and the disulfide bonds formed can be decomposed by intracellular GSH, which exhibits useful application prospects in the preparation of GSH-responsive hydrogels. Moreover, thiolated HA can effectively improve the cell-adhesion ability of hydrogels when mixed with peptides and collagen. Phenolized HA is synthesized *via* an acylation reaction using Tyr, Dopa, and polyphenols to graft HA molecules. Phenolized HA-based hydrogels have been prepared primarily through radical coupling reactions, polyphenol-metal coordination, and polyphenol-boronate complexation. Dopa- and polyphenol-modified HA-based hydrogels exhibit good tissue adhesion and cell affinity owing to the presence of catechol groups (mussel biomimetic chemistry), which can reduce cartilage friction and wear to protect cartilage. In addition, many polyphenol-modified HA-based hydrogels can inhibit inflammation owing to their antioxidant and anti-inflammatory properties, which provide useful applications in OA therapy. Hydrazide HA is synthesized primarily using ADH to modify HA through an acylation reaction. Hydrazide HA has abundant hydrazides and can react with polymers containing aldehyde groups through a Schiff-base reaction to develop hydrogels. Host–guest group-modified HA-based hydrogels are prepared using host or guest modification of HA and polymers containing host or guest groups through host–guest interactions. Owing to the dynamic reversibility of both the Schiff-base reaction and host–guest interaction, these two types of hydrogels exhibit the desirable merits of mild preparation conditions, rapid gelation time, good self-healing capacity, and sustained release of bioactive substances.

Furthermore, we reviewed modified HA-based hydrogels for 3D bioprinting in cartilage tissue engineering applications. 3D bioprinted hydrogels generally have double crosslinking networks involving dynamic click chemistry crosslinking and photocrosslinking. Primary dynamic crosslinking endows hydrogels with injectable and self-healing abilities, and further photocrosslinking effectively enhances the mechanical properties and stability of hydrogels. Although tremendous progress has been achieved in HA-based hydrogels for cartilage tissue engineering, current cartilage repair studies are limited to small animals, and only a few reports for large animals are available. In addition, further clinical applications of HA-based hydrogels still encounter significant challenges, such as undetermined molecular weight distribution and inaccurate chemical structure of polymers, inflammatory responses, degeneration effects of proteins, and generation and activation of hyaluronidase. In conclusion, at the intersection of materials science and regenerative medicine, we believe that multifunctional HA-based hydrogels with high biomimicking of the cartilage ECM will play an important role in cartilage tissue engineering. HA-based hydrogels are a class of products with significant clinical application value in the near future.

## Author contributions

Min Wang: Investigation, Writing – original draft, Visualization. Zexing Deng: Investigation, Writing – original draft. Yi Guo: Investigation, Writing-editing. Peng Xu: Supervision, Conceptualization.

## Funding

We thank the financial support of the 10.13039/501100002858China Postdoctoral Science Foundation (grant No. 2021M702644), the 10.13039/501100007128Natural Science Foundation of Shaanxi Province (grant No. 2022JQ-384) and the High-level Talents Foundation for Scientific Research of Xi'an University of Science and Technology (grant No. 2050122015).

## Declaration of competing interest

The authors declare that they have no known competing financial interests or personal relationships that could have appeared to influence the work reported in this paper.

## Data Availability

The authors have obtained figures permission.
